# A passive mechanically tunable conformal aperture coupled phase shifter integrated with micron-sized magnetic particles for radar systems

**DOI:** 10.1038/s41598-026-53679-x

**Published:** 2026-05-19

**Authors:** Muhammad Ayaz, Ahmed Alqurashi, Turki Essa Alharbi, Fatima Abdul Rasheed

**Affiliations:** 1 Department of Electronics Engineering, UET Peshawar Abbottabad Campus, Abbottabad, 22020 Pakistan; 2 Department of Electrical Engineering, College of Engineering and Architecture, Umm Al-Qura University, Makkah, 24382 Saudi Arabia; 3 Department of Electrical Engineering, College of Engineering, Taif University, Taif, 21944 Saudi Arabia

**Keywords:** Phase shifter, Conformal, Magneto static responsive structures, Aperture coupled stubs, Loading stubs, Antenna, Beamforming, Engineering, Physics

## Abstract

This work presents a conformal Micron-Sized Magnetic Particle Aperture-Coupled (µMP-AC) phase shifter for C-band (7.2–8.2 GHz) applications, implemented on a flexible Rogers RT5880 substrate. Unlike conventional phase shifters that rely on complex electrical biasing networks, the proposed design employs a purely passive tuning mechanism based on Magnetic Induction–Responsive Cavities (MIRCs). These MIRCs are embedded within the flexible substrate in concentric ring configurations in the aperture-coupled region. By mechanically moving a single small magnet, the effective permittivity of the particle-loaded substrate varies, enabling controlled linear phase shifts of 0°, 20°, and 40° on both planar and cylindrically curved surfaces (*R* = 100 mm). The proposed µMP-AC phase shifter is comprehensively validated through full-wave electromagnetic simulations, equivalent circuit modeling, and experimental measurements conducted in an anechoic chamber. Experimental results show close agreement with the equivalent circuit model results, demonstrating an insertion loss better than 5 dB and a return loss of 37.24 dB at 7.3 GHz. The measured phase error using Network Analyzer is approximately 1.2°, confirming stable linear phase behavior. By eliminating external DC biasing circuits, the proposed µMP-AC phase shifter offers a compact, low-complexity solution for conformal radar front ends and beam-steering systems.

## Introduction

Conformal and low-profile RF components are increasingly essential for satellite communication, modern radar, and wearable applications, where antenna arrays and phase-shifting networks must conform to non-planar or flexible surfaces^1–5^. Using conformal phase shifters, the electromagnetic radiation can be controlled and steered while keeping aerodynamic profiles, reducing surface protrusion, and making antenna arrays lighter^5^. Such RF components make it easy to integrate them into fuselages, vehicle roofs, satellite platforms, and wearable systems. These components need to be mechanically strong and sufficiently compact to remain stable when bent or curved in RF system.

Furthermore, microwave systems operating in the C-band require more than just mechanical conformity. They also require a wide bandwidth, low insertion loss, stable phase coverage, and good impedance matching for application. Add functional materials like magnetically responsive micro-sized particles or magnetoelectric composites provide additional electromagnetic tunability and performance enhancement. For conformal surface applications, compact multilayer topologies, including aperture coupling and stub loading, are particularly attractive as they support miniaturization while maintaining favorable RF trade-offs^6^.

In recent literature, the researchers addressed some main challenges in the development of conformal phase shifters and flexible phased arrays. In^1^, multiple fabrication techniques for flexible substrates, such as inkjet printing and additive manufacturing, are studied alongside with their applications in conformal phased arrays. These arrays have been designed on singly curved, cylindrical, and spherical surfaces, with phase control accomplished using composite right/left-handed (CRLH) transmission lines^7–9^. These works also explored the impact of mechanical bending on element phasing (deg) and propose methods to improve mechanical robustness and on-surface integration^7–9^. Furthermore, a flexible phased array incorporating self-calibration and printed transceiver integration was studied in^10^, presenting practical strategies to preserve radiation performance under surface deformation. Real-time deformation correction and additive manufacturing techniques have also been investigated to minimize fabrication and bending-induced errors in printed conformal arrays^11^.

To achieve large and continuous delays, aperture-coupled and multilayer slot-coupling techniques have long been used in reflect array and phased-array designs applications. The author in^12^ presented an aperture-coupled reflect array elements capable of providing wide phase-delay ranges using true-time-delay lines and multilayer coupling, establishing aperture coupling as a fundamental topology for achieving extensive phase control in printed multilayer structures. Further detailed study of aperture- and slot-coupled unit cells have introduced circuit-level models and practical multilayer layouts directly applicable to printed phase-shifter designs^13,14^. Such approaches are commonly used stub-loaded and stub-coupled configurations to support wideband phase-shifting operation. Additional investigations on aperture-coupled resonators and multi-section stub networks emphasize the importance of precise stub placement and coupling control in achieving ultra-wideband phase shifts, high selectivity, and improved overall performance^15–17^. Reflection-type aperture-coupled phase shifters and hybrid-coupler-based reflective designs further extend the available design space for wide phase shift and tunable RF systems^17,18^.

Some material based tuning approaches have also been widely studied for microwave devices, including magnetoelectric composites, ferrite or ferroelectric thin films, and flexible polymers embedded with magnetic particles. Using external magnetic or electric fields, the resonance frequency and phase response can be efficiently controlled^19,20^. Further research on magnetic composites explored that particle size, concentration, and spatial distribution significantly affect the effective permeability, permittivity, microwave loss, and resonance characteristics^21,22^. When embedding magnetic particles to printed RF system, such as phased antenna arrays, these results offer helpful guidance for striking a balance between tunability and loss (dB). The EM behavior and fabrication techniques of particle-embedded microwave materials have been thoroughly studied in^3,7,23^.The efficiency of particle-enhanced flexible materials for tunable microwave devices has been studied in last years by the proposal of conformal phase shifters based on CRLH transmission lines with embedded magnetic microparticles to achieve phase control (degrees) on conformal surfaces^7^. Concurrently, low-loss (dB) phase shifters with mechanically flexible configurations operating in a wide frequency ranges (GHz) have been explored by substrate integrated waveguide (SIW) and gap-waveguide technologies, providing helpful benchmarks in terms of insertion loss (dB) and achievable phase range^25–29^. By addressing practical challenges like surface deformation, additive manufacturing, and the impact of mechanical curvature on EM performance, these articles collectively advance the development of conformal, particle-embedded printed phase shifters.

Regardless of these explorations, most multilayer aperture-coupled phase shifters currently in use mainly rely on electrical biasing methods for phase tuning. To achieve precise and continuous phase control via magnetic biasing in conformal, aperture-coupled printed structures still have a significant gap in literature. Magnetoelectric films and particle composites based on ferrite or magnetite are examples of emerging materials that present new possibilities for the implementation of magnetically tunable phase shifters with straightforward biasing schemes. Nevertheless, a unified design that incorporates low insertion loss, particle-enhanced materials, aperture coupling, conformality, and ease of fabrication has not yet been fully achieved.

Recently, several magnetically and electrically tunable phase shifters have been reported. For example, SIW-based phase shifters such as^30^ provide compact implementations using slow-wave structures, while magnetoelectric-based designs such as^31^ employ ferrite/PZT thin films with electrical biasing for tunability. However, these approaches typically require complex biasing networks, rigid structures, or continuous material layers. In contrast, the proposed µMP-AC phase shifter introduces a bias-free tuning mechanism using a single movable magnet and discrete particle-loaded cavities. Furthermore, the proposed design supports conformal implementation on curved surfaces, which is not addressed in these works.

This work introduces a conformal aperture-coupled phase shifter with micron-sized magnetic particles to close this gap. This design, called the Micron-Sized Magnetic Particle Aperture-Coupled (µMP-AC) phase shifter, is driven by the lack of an integrated solution that combines particle-based magnetic tuning, aperture coupling, and conformal geometry, as well as the increasing need for compact, tunable microwave components. Circular aperture-coupled stubs made on a flexible Rogers RT5880 substrate are used in the µMP-AC phase shifter to provide stable electromagnetic performance and mechanical flexibility. Newly developed micron-sized magnetic particles^32–39^ are embedded between the upper and lower circular stubs in a concentric configuration to enable magnetic tunability. A small external magnet, providing a magnetic field strength exceeding 50 mT, is positioned beneath the stub region to achieve precise phase control. By simply moving this single tiny magnet to different radial positions beneath the lower stub, accurate, linear and continuous phase shifting is achieved in the frequency range 7.2–8.2 GHz.

The key novelties of this work are as follows: (1) the entire phase-shifting operation is controlled using a single magnet; (2) external DC biasing circuits are completely eliminated; (3) the proposed µMP-AC phase shifter is compatible with both planar and conformal cylindrical surfaces; (4) it operates over a wide bandwidth of 1 GHz (7.2–8.2 GHz) with moderate insertion loss; and (5) unlike conventional SIW or magnetoelectric thin-film phase shifters, the proposed design employs discrete particle-loaded cavities with a fully passive magnetic tuning mechanism, thereby eliminating the need for DC biasing networks.

Furthermore, unlike conventional electrically biased approaches, the µMP-AC phase shifter achieves continuous and precise phase control through the simple mechanical movement of a single tiny magnet, offering a compact, moderate insertion loss, and practical solution for conformal phase-shifting applications, particularly in radar systems.

Further, this paper is organized as follows. Section II describes the configuration and electromagnetic operation of the µMP-AC phase shifter. Full-wave simulation results for the planar configuration are presented in Section III, followed by conformal surface results in Section IV. Section V discusses the development and validation of an equivalent circuit model. Measurement results and experimental validation are presented in Section VI, and conclusion is given in Section VII.

## Configuration and electromagnetic operation of a magnetically controlled µMP-AC phase shifter

**Fig. 1:**
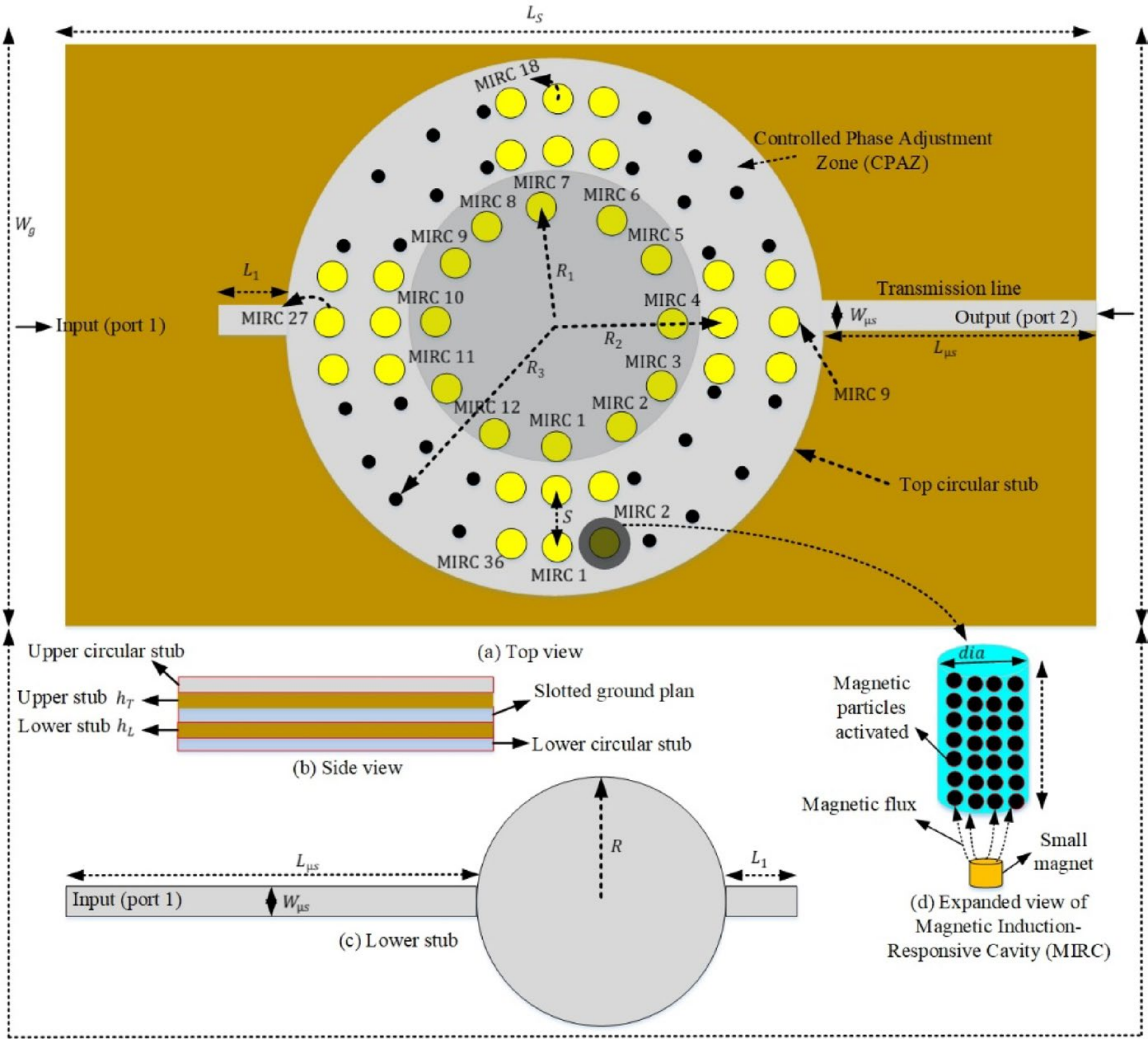
Geometry of the planar µMP-AC phase shifter (**a**) top view consists of upper substrate, circular stub and transmission line (**b**) side view (**c**) lower circular stub of radius R (**d**) magnetic induction-responsive cavity structure.

The proposed Micron-Sized Magnetic Particle–based Aperture-Coupled (µMP-AC) phase shifter, illustrated in Fig. 1, is a planar aperture-coupled structure designed to achieve tunable phase control using a single tiny magnet. The design incorporates two circular stubs, an upper and a lower stub, each having a radius R. The upper circular stub and the Rogers RT5880 substrate are drilled with circular cavities arranged in three concentric configurations with radii R₁ = 3 mm, R₂ = 6 mm, and R₃ = 9 mm. The R₁ configuration contains 12 cavities, the R₂ configuration contains 24 cavities, and the R₃ configuration contains 36 cavities. Each cavity is drilled with diameter 1 mm. Further, both stub layers are implemented on flexible Rogers RT5880 substrates with a relative permittivity of 2.2, a loss tangent of 0.0009, and a thickness of $\:{\mathrm{h}}_{\mathrm{T}}={\mathrm{h}}_{\mathrm{L}}=0.787\:\mathrm{m}\mathrm{m}$, where $\:{\mathrm{h}}_{\mathrm{T}}$and $\:{\mathrm{h}}_{\mathrm{L}}$ denote the thicknesses of the upper and lower substrates, respectively. The upper substrate supports a microstrip transmission line with width $\:{\mathrm{W}}_{{\upmu\:}\mathrm{s}}$ and length $\:{\mathrm{L}}_{{\upmu\:}\mathrm{s}}$, which is connected to the upper circular stub and defines the Controlled Phase Adjustment Zone (CPAZ**)**, the primary region responsible for phase modulation. A matching microstrip line with identical dimensions (width $\:{\mathrm{W}}_{{\upmu\:}\mathrm{s}}$ and length $\:{\mathrm{L}}_{{\upmu\:}\mathrm{s}}$ is designed on the lower substrate and connected to the lower circular stub, as shown in Fig. 1c. A slotted ground plane is placed between the two substrates, featuring a slot width of 12 mm and a slot length of 24 mm, enabling efficient aperture coupling between the upper and lower stub layers.

Here, the electromagnetic (EM) operational behavior and working principle of the proposed µMP-AC phase shifter are explained in detail. To achieve a precisely controlled phase response, a Controlled Phase Adjustment Zone (CPAZ) is introduced shown in Fig. 1, consisting of an array of circularly arranged cavities. Each cavity in the flexible substrate is partially filled with micron-sized magnetic particles^30^, forming localized regions that respond to external magnetic excitation. When a tiny magnet, as illustrated in Fig. 1(d), is placed beneath the CPAZ region, these magnetic particles become magnetically aligned in vertical columns, which modifies the effective permittivity of the substrate in the cavity area^37^. This controlled variation in electromagnetic properties enables a precise, repeatable, and bias-free phase shift across the desired frequency band (7.2–8.2) GHz. Because of this magnetic induction-based tuning mechanism, each cavity is referred to as a Magnetic Induction-Responsive Cavity (MIRC). The circular array at radius R₁ comprises of MIRC 1 to MIRC 12 cavities and serves as the reference MIRC array, providing the same phase response for all MIRC activations over the operating frequency band (7.2–8.2) GHz.

The 2nd MIRC array positioned at R_2_ consists of MIRC 1 to MIRC 24 cavities, it gives the maximum phase response up to $\:{20}^{^\circ\:}$. Furthermore, a third circular configuration containing MIRC 1 to MIRC 36 cavities is embedded at radius R_3_, achieving a maximum phase response of up to $\:{40}^{^\circ\:}$. Moreover, the phase-shift range can be further extended through cascaded configurations of µMP-AC units. The key significance of this approach lies in its simplicity and efficiency. By moving a single small magnet beneath any of the circular regions R_1_, R_2_ or R_3_, the desired phase shift can be achieved without any external DC biasing circuits, which are typically required in conventional designs. This makes the µMP-AC architecture lightweight, low-cost, and easy to reconfigure, while maintaining high reliability and flexibility for conformal C-band systems. In next section, detailed full wave simulation results of all three array configurations are presented on planar and conformal surfaces.

## Full-wave electromagnetic simulation results of the conformal µMP-AC phase shifter

### Planar µMP-AC phase shifter

In this section, detailed full-wave Electromagnetic (EM) simulation results (using CST Microwave Studio Suite 2025) of the proposed µMP-AC phase shifter shown in Fig. 1 are presented for the planar configuration. The design is evaluated for all achievable phase shifts (deg) by sequentially activating individual MIRCs in each of the three circular configurations with radii *R₁*, *R₂*, and *R₃*. For all MIRC activations, the reflection coefficient satisfies $\:{\left|S\right|}_{11}\le\:-10\:\mathrm{d}\mathrm{B}$ over the entire operating band, while the insertion loss remains better than ($\:\left|{S}_{21}\right|$ $\:<-5\:\mathrm{d}\mathrm{B})$. Moreover, the phase response in all three configurations exhibits linear behavior across the full frequency range of 7.2–8.2 GHz, indicating stable and broadband phase control.

**Fig. 2:**
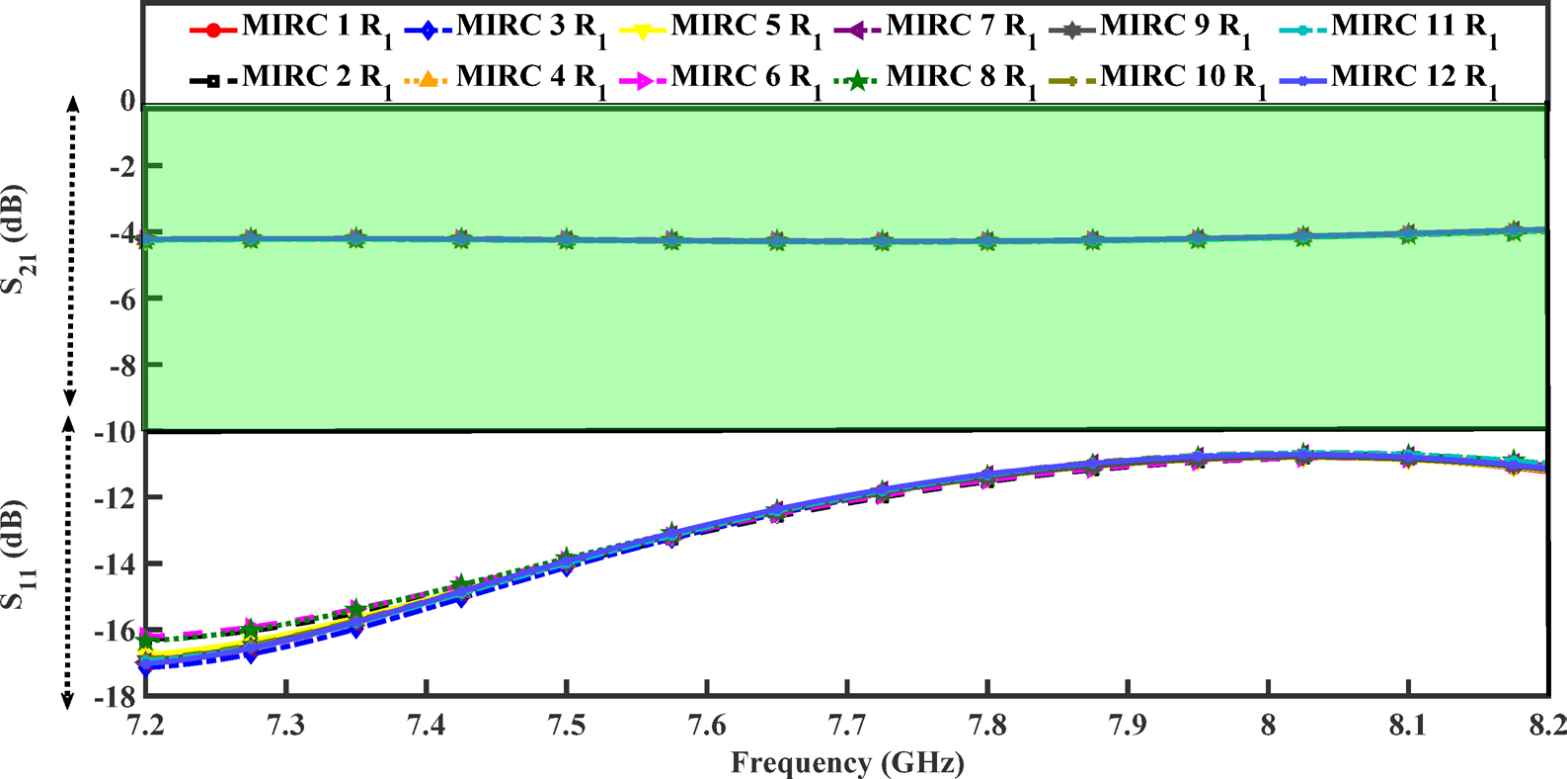
µMP-AC phase shifter reflection $\:\left|{S}_{11}\right|$ and transmission $\:\left|{S}_{21}\right|$ coefficients in dB for sequential activation of MIRCs (1–12) in reference array at $\:{\mathrm{R}}_{1}=3\:\mathrm{m}\mathrm{m}$.

**Fig. 3:**
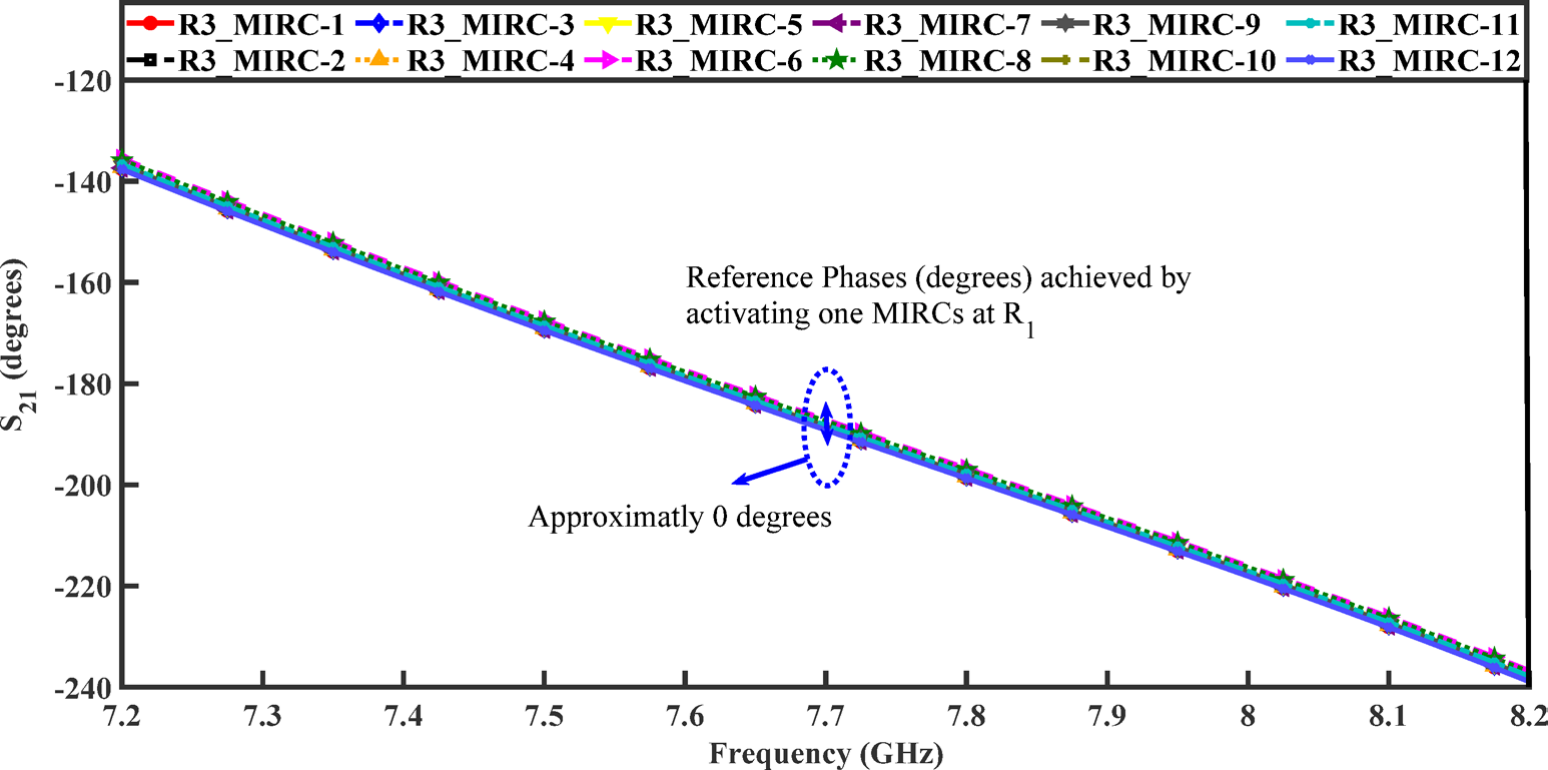
µMP-AC transmission phase response $\:{S}_{21}{\left(\right)}^{^\circ\:}$, for sequential activation of MIRCs (1–12) in the reference array at radius at $\:{\mathrm{R}}_{1}=3\:\mathrm{m}\mathrm{m}$.

First, the twelve MIRCs positioned at radius R₁ = 3 mm are sequentially magnetically activated using a single tiny magnet with a diameter of 1 mm placed beneath the lower substrate. The simulated reflection coefficients $\:{\left|S\right|}_{11}$ and insertion loss $\:\left|{S}_{21}\right|$ in dB for all MIRC activations at R₁ are shown in Fig. 2, while the corresponding transmission phase response ∠S_21_, is presented in Fig. 3. The results demonstrate that, irrespective of which MIRC is activated, the phase response remains identical and linear across the entire frequency band. At the center frequency of 7.7 GHz, the phase is approximately $\:{0}^{^\circ\:},$ and therefore the MIRC configuration at radius R₁ is designated as the reference MIRC array.

To further extend the transmission phase response $\:{S}_{21}{\left(\right)}^{^\circ\:}$ beyond 0° (shown in Fig. 3 at $\:{\mathrm{R}}_{1}=3\:\mathrm{m}\mathrm{m})$, an additional circular array of MIRCs is implemented at a radius of $\:{\mathrm{R}}_{2}=6\:\mathrm{m}\mathrm{m}$, comprising 24 MIRCs. Each MIRC in this configuration is sequentially activated and evaluated in terms of reflection coefficient $\:\left|{S}_{11}\right|$, insertion loss $\:\left|{S}_{21}\right|$ in dB and transmission phase response $\:{S}_{21}{\left(\right)}^{^\circ\:}$.

**Fig. 4:**
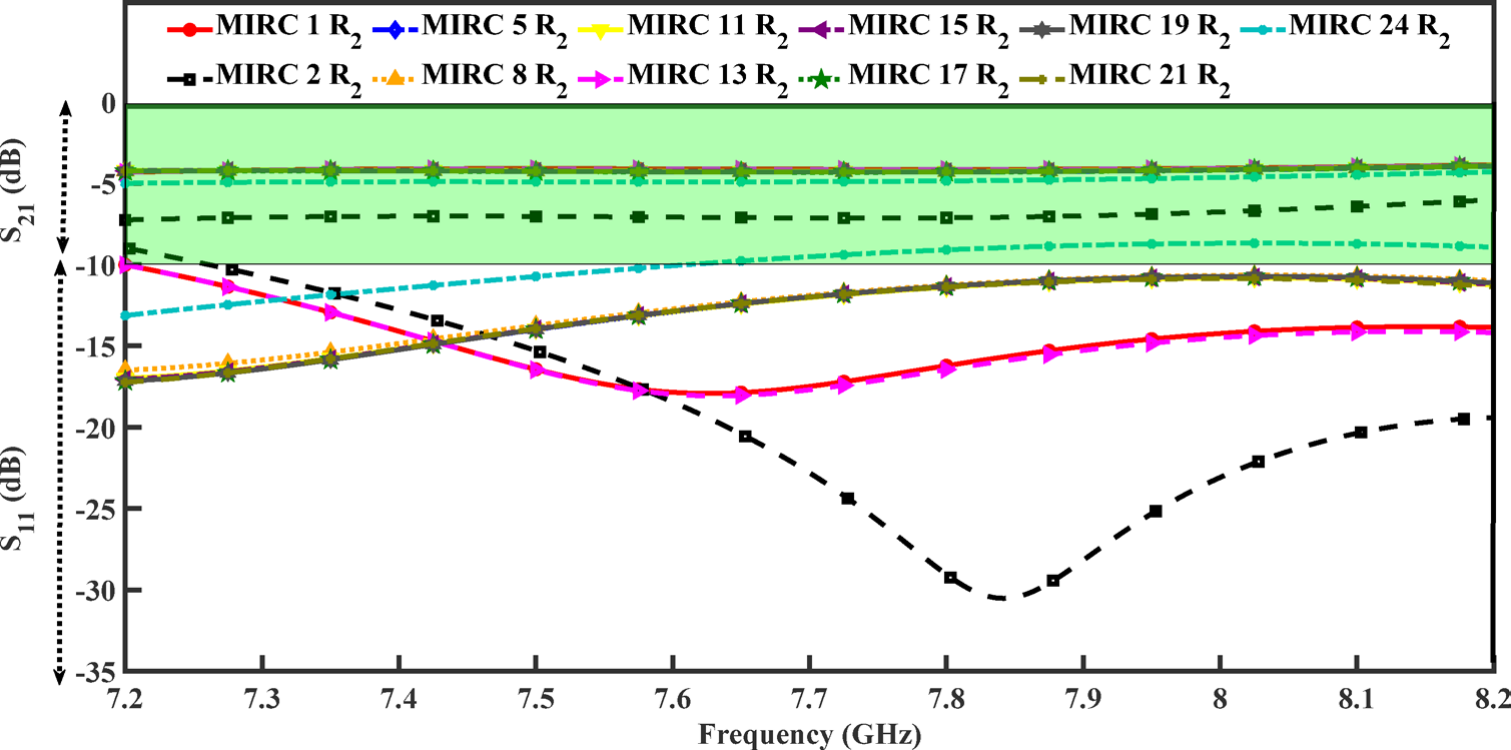
µMP-AC phase shifter reflection $\:\left|{S}_{11}\right|$ and transmission $\:\left|{S}_{21}\right|$ coefficients in dB for sequential activation of MIRCs (1–24) in reference array at $\:{\mathrm{R}}_{2}=6\:\mathrm{m}\mathrm{m}$.

**Fig. 5:**
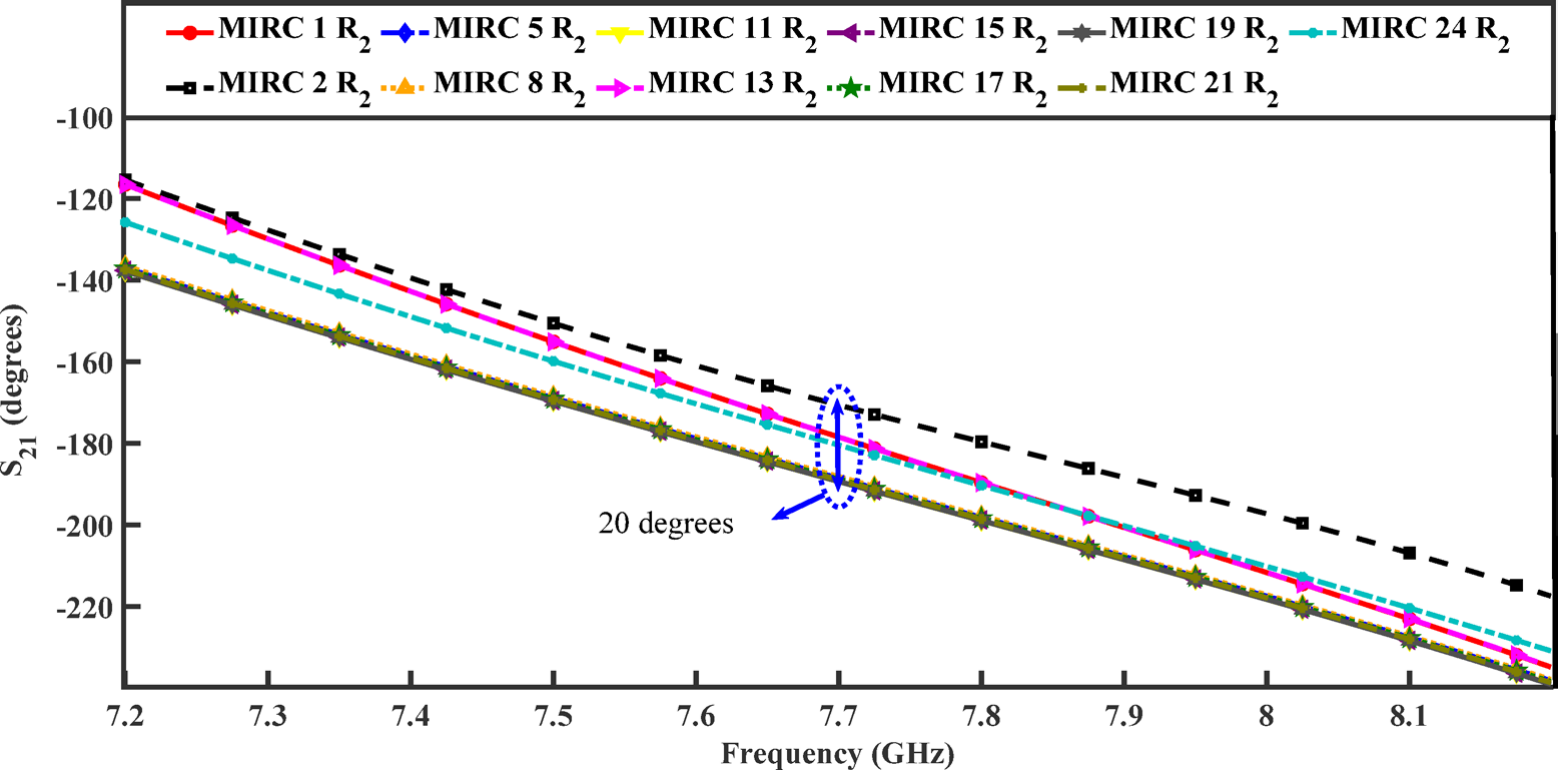
µMP-AC transmission phase response $\:{S}_{21}{\left(\right)}^{^\circ\:}$, for sequential activation of MIRCs (1–24) in the reference array at radius at $\:{\mathrm{R}}_{2}=6\:\mathrm{m}\mathrm{m}$.

The simulated $\:\left|{S}_{11}\right|$, and $\:\left|{S}_{21}\right|$ results are presented in Fig. 4, demonstrating that the proposed µMP-AC phase shifter operates efficiently over the entire frequency band of 7.2–8.2 GHz, with $\:{\left|S\right|}_{11}\le\:-10\:\mathrm{d}\mathrm{B}$ and insertion loss consistently better than ($\:\left|{S}_{21}\right|$ $\:<-5\:\mathrm{d}\mathrm{B})$ for all MIRC activations. In parallel, the corresponding phase responses are illustrated in Fig. 5.

The results confirm that, by activating individual MIRCs in the circular array at $\:{\mathrm{R}}_{2}=6\:\mathrm{m}\mathrm{m}$, the transmission phase $\:{S}_{21}{\left(\right)}^{^\circ\:}$ is extended $\:{20}^{^\circ\:}$in a controlled and continuous manner, thereby enabling phase progression beyond the reference state established at $\:{\mathrm{R}}_{1}$. This extended phase coverage verifies the effectiveness of the $\:{\mathrm{R}}_{2}$ MIRC array in providing enhanced phase control while maintaining broadband impedance matching and moderate insertion loss performance.

Building on the phase extension achieved with the $\:{\mathrm{R}}_{2}$ MIRCs configuration, a third circular array of MIRCs is introduced at a larger radius of $\:{\mathrm{R}}_{3}=9\:\mathrm{m}\mathrm{m}$ to further increase the achievable phase range$\:\:{S}_{21}{\left(\right)}^{^\circ\:}$.This array consists of 36 MIRCs, each of which is magnetically activated individually to evaluate its impact on the electromagnetic performance of the µMP-AC phase shifter. Like the previous cases shown from Figs. 2, 3, 4 and 5, the reflection coefficient ∣$\:\left|{S}_{11}\right|$, insertion loss $\:\left|{S}_{21}\right|,$ and transmission phase response$\:\:{S}_{21}{\left(\right)}^{^\circ\:}$are analyzed for each MIRC activation across the operating frequency band 7.2–8.2 GHz.

**Fig. 6:**
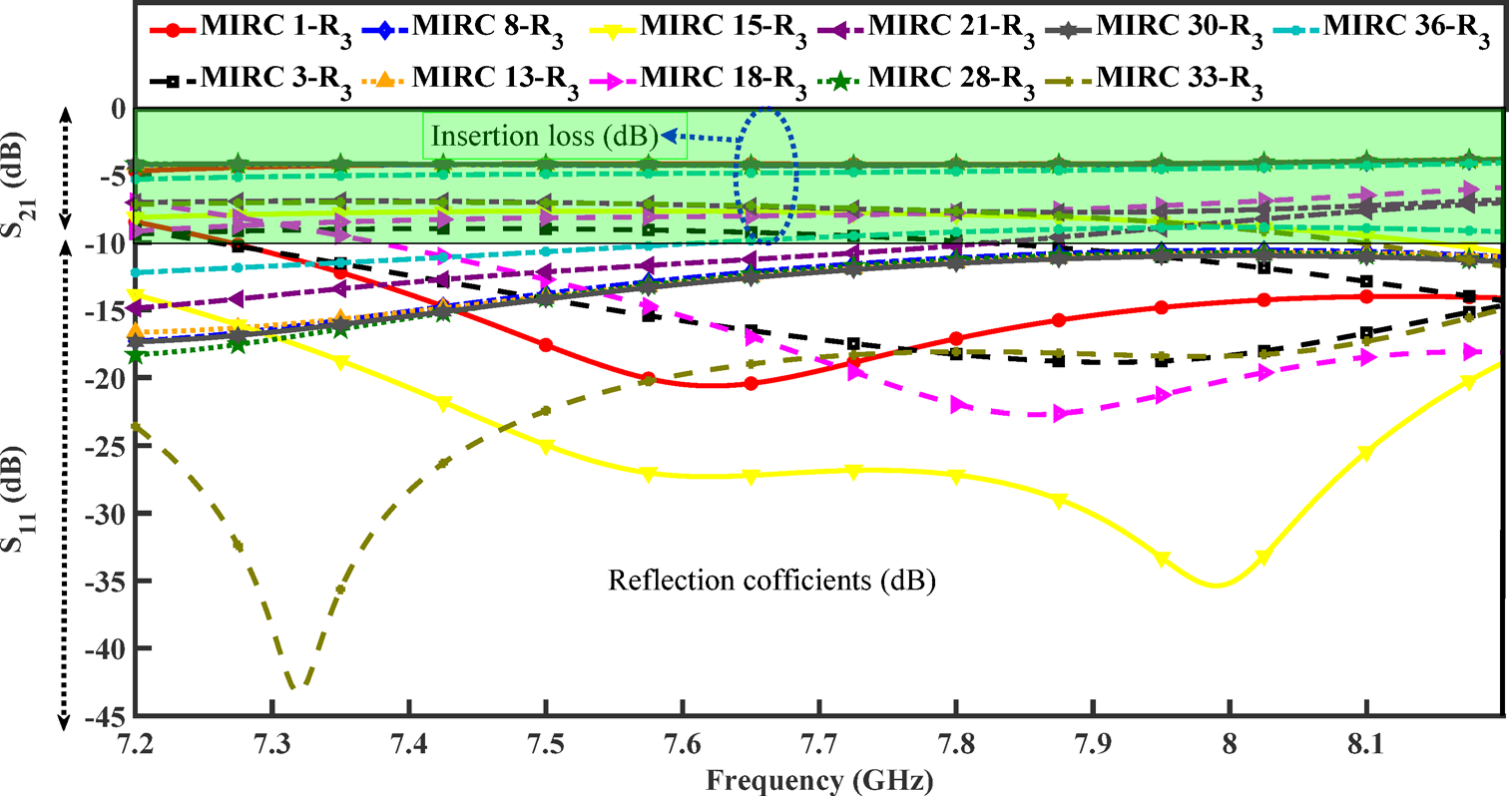
µMP-AC phase shifter reflection $\:\left|{S}_{11}\right|$ and transmission $\:\left|{S}_{21}\right|$ coefficients in dB for sequential activation of MIRCs (1–36) in reference array at $\:{\mathrm{R}}_{3}=9\:\mathrm{m}\mathrm{m}$.

**Fig. 7:**
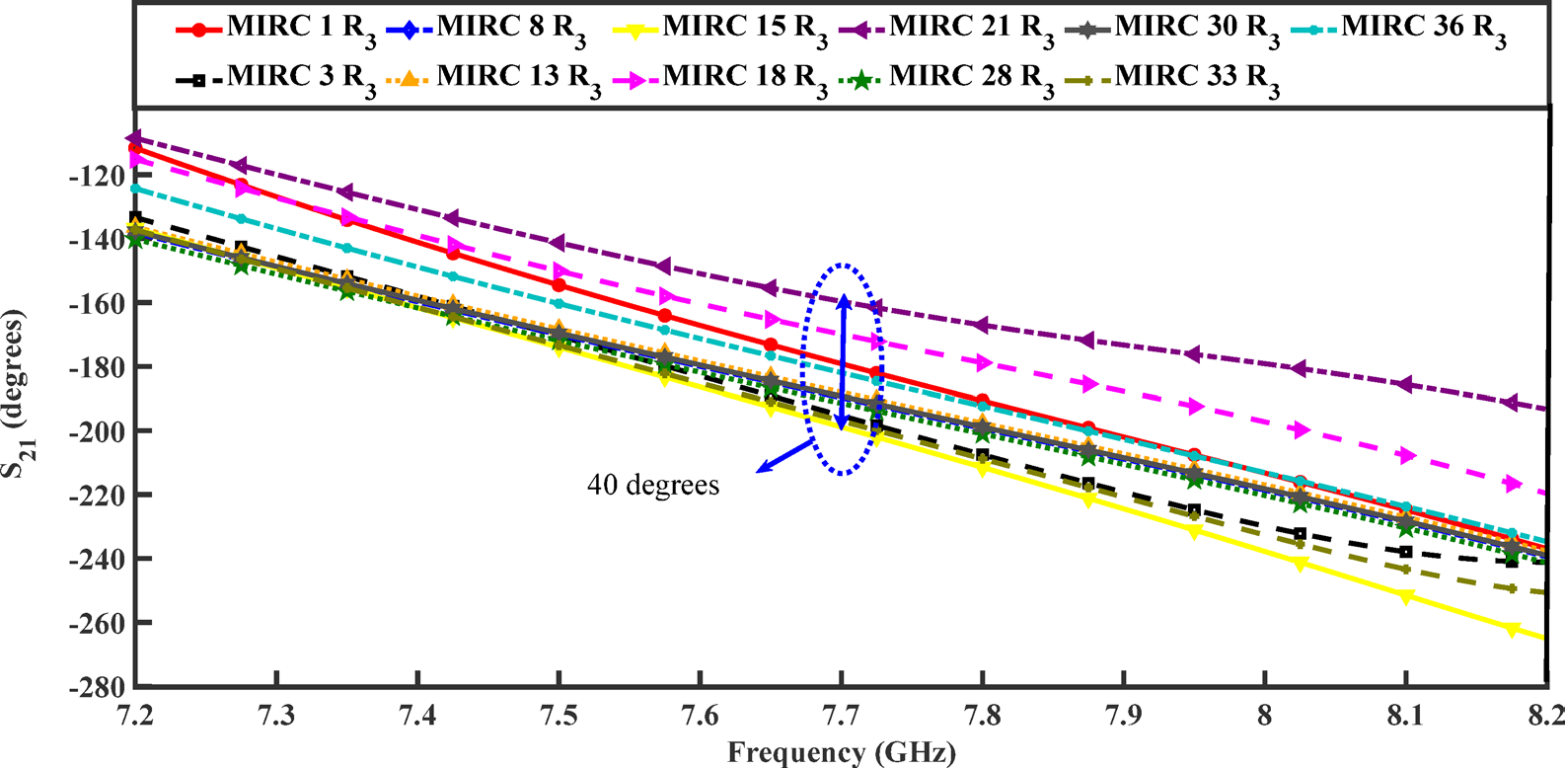
µMP-AC transmission phase response $\:{S}_{21}{\left(\right)}^{^\circ\:}$, for sequential activation of MIRCs (1–36) in the reference array at radius at $\:{\mathrm{R}}_{3}=9\:\mathrm{m}\mathrm{m}$.

The full wave simulated $\:\left|{S}_{11}\right|\:$and $\:\left|{S}_{21}\right|\:$results (shown in Fig. 6) confirm that the proposed µMP-AC phase shifter maintains stable and efficient operation over the entire 7.2–8.2 GHz frequency range, with $\:{\left|S\right|}_{11}\le\:-10\:\mathrm{d}\mathrm{B}$ and insertion loss better than ($\:\left|{S}_{21}\right|$ $\:<-5\:\mathrm{d}\mathrm{B})$ for all MIRCs in the $\:{\mathrm{R}}_{3}\:$array. The corresponding phase responses (in Fig. 7) demonstrate a further extension of the transmission phase $\:{S}_{21}{\left(\right)}^{^\circ\:}$, achieving a maximum phase shift of approximately 40° at the center frequency. This additional phase coverage, enabled by the larger radial placement of the MIRCs at $\:{\mathrm{R}}_{3}\:$highlights the scalability of the proposed approach and confirms that progressive phase tuning can be realized by appropriately selecting the radial position of the magnetically activated MIRCs while preserving broadband and moderate insertion loss characteristics.

From the above full-wave electromagnetic simulation results of the proposed µMP-AC phase shifter demonstrate that a desired transmission phase $\:{S}_{21}{\left(\right)}^{^\circ\:}$, can be achieved by simply using a single tiny magnet beneath the lower substrate along circular trajectories (R_1_-R_3_). A reference phase state of approximately 0° is obtained when the magnet is positioned at radius R_1_ = 3 mm. By moving the magnet in counterclockwise direction at radius R₂ = 6 mm, the achievable phase range is extended up to approximately 20°, while further moving to radius R₃ = 9 mm enables phase shifts of up to approximately 40°. The µMP-AC phase range can be further increased through two possible scalability approaches: (i) placing the magnet at larger radial positions (e.g., 12, 15, or 18 mm) or (ii) cascading multiple µMP-AC unit-cell configurations.

Next, the proposed µMP-AC phase shifter is evaluated in terms of root-mean-square (RMS) phase and magnitude responses.

**Fig. 8:**
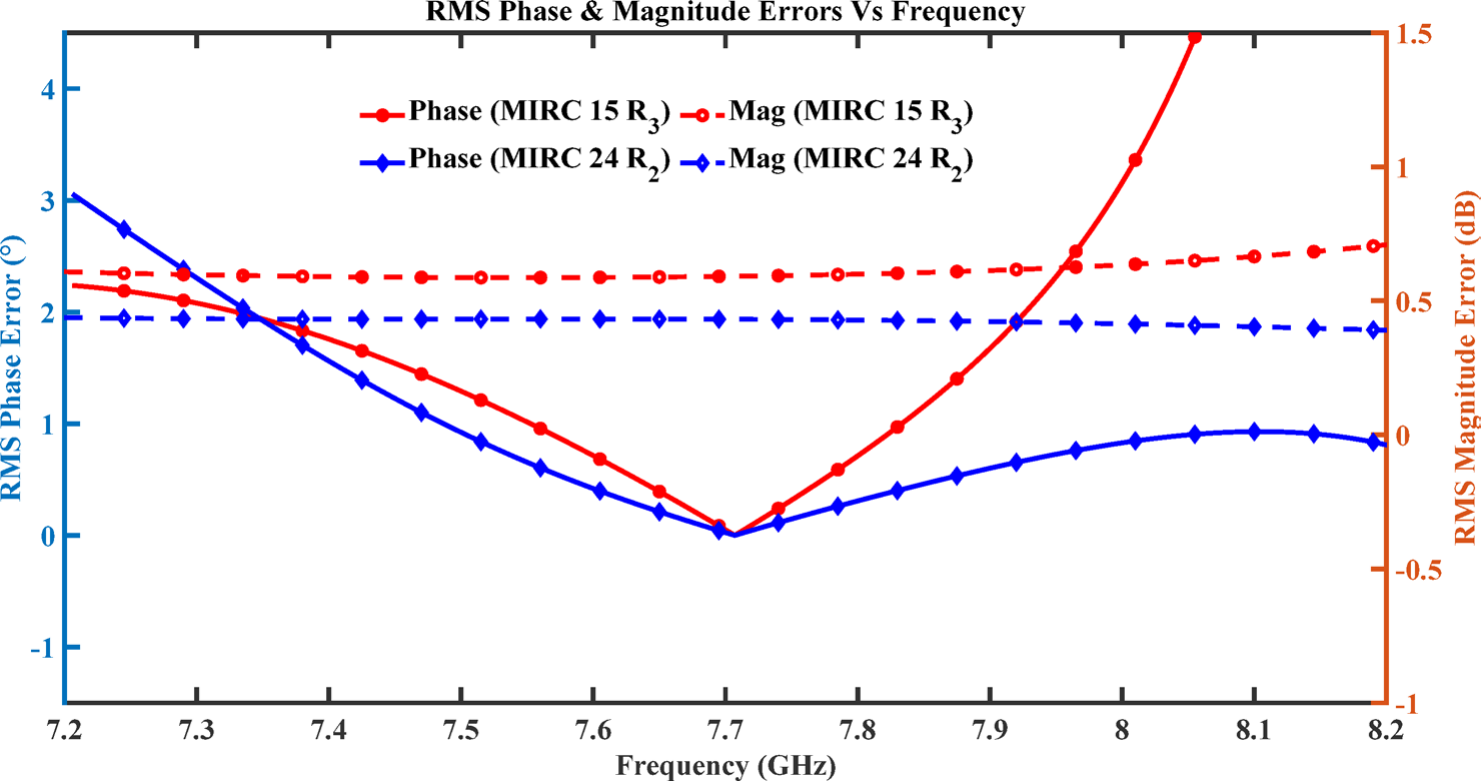
µMP-AC phase shifter RMS phase $\:{\left(\right)}^{^\circ\:}$ and magnitude error for sequential activation of MIRCs in the reference array at radius at $\:{\mathrm{R}}_{2}$ and $\:{\mathrm{R}}_{3}$.

Since the phase response at R₁ corresponds to the reference state and remains approximately 0°, the RMS performance metrics are analyzed for the MIRC activations at radii R₂ and R₃. Figure 8 presents the RMS phase response, which remains within 0–5° across the entire operating frequency band of 7.2–8.2 GHz for all MIRC activations. This indicates good phase of stability and consistency over the bandwidth. Similarly, the RMS magnitude response is observed to lie between 1 and 2.5 dB. It is noted that the RMS magnitude error increases slightly as the MIRCs are activated at larger radial positions. This behavior is attributed to stronger electromagnetic perturbations introduced by the magnetically activated particles at increased radii; however, the magnitude variations remain within acceptable limits and do not significantly degrade the overall performance of the phase shifter.

From the above EM simulation results of the proposed µMP-AC phase shifter in terms of reflection $\:\left|{S}_{11}\right|$, transmission $\:\left|{S}_{21}\right|$ coefficients, phase response $\:{S}_{21}{\left(\right)}^{^\circ\:}$, RMS phase error $\:{\left(\right)}^{^\circ\:}$ and RMS magnitude error (dB) it can be concluded that, the design introduces a highly novel approach to achieve phase shift. This mechanism enables continuous phase tuning using a single movable tiny magnet, with selected discrete phase states demonstrated. This approach completely eliminates the need for multiple magnets and external DC biasing circuits commonly required in conventional phase shifters, significantly simplifying the system architecture. The proposed technique is therefore well suited for compact and conformal phase-shifting applications, particularly in radar systems. The next section validates the performance of the proposed phase shifter on a cylindrical conformal geometry.

### Conformal µMP-AC phase shifter

In this section, full-wave electromagnetic simulation results of the proposed µMP-AC phase shifter are investigated for operation on a cylindrical conformal surface with a radius of 100 mm. For accurate full-wave modeling, the Magnetic Induction-Responsive Cavities (MIRCs) are implemented in CST Microwave Studio by first defining cylindrical cavities drilled into the substrate. These cavities are assumed to be filled with micro-sized silver-coated magnetic particles, which are stacked vertically to form a cylindrical particle-loaded region. In the simulation, this particle-filled structure is modeled as an equivalent homogeneous material whose effective permittivity represents the collective behavior of the particles under magnetic excitation. The variation in effective permittivity emulates the alignment of particles when subjected to an external magnetic field, enabling accurate representation of the phase tuning mechanism without explicitly modeling individual particles. In the planar configuration, three circular MIRC arrays located at radii R₁, R₂, and R*₃* were considered. However, as investigated earlier, activation of any MIRC at R_1_ consistently provides a reference phase shift of approximately 0°. Therefore, for the conformal analysis, only the MIRC activations at radii R_2_ and R_3_ are evaluated, as these configurations are responsible for linear phase response.

**Fig. 9:**
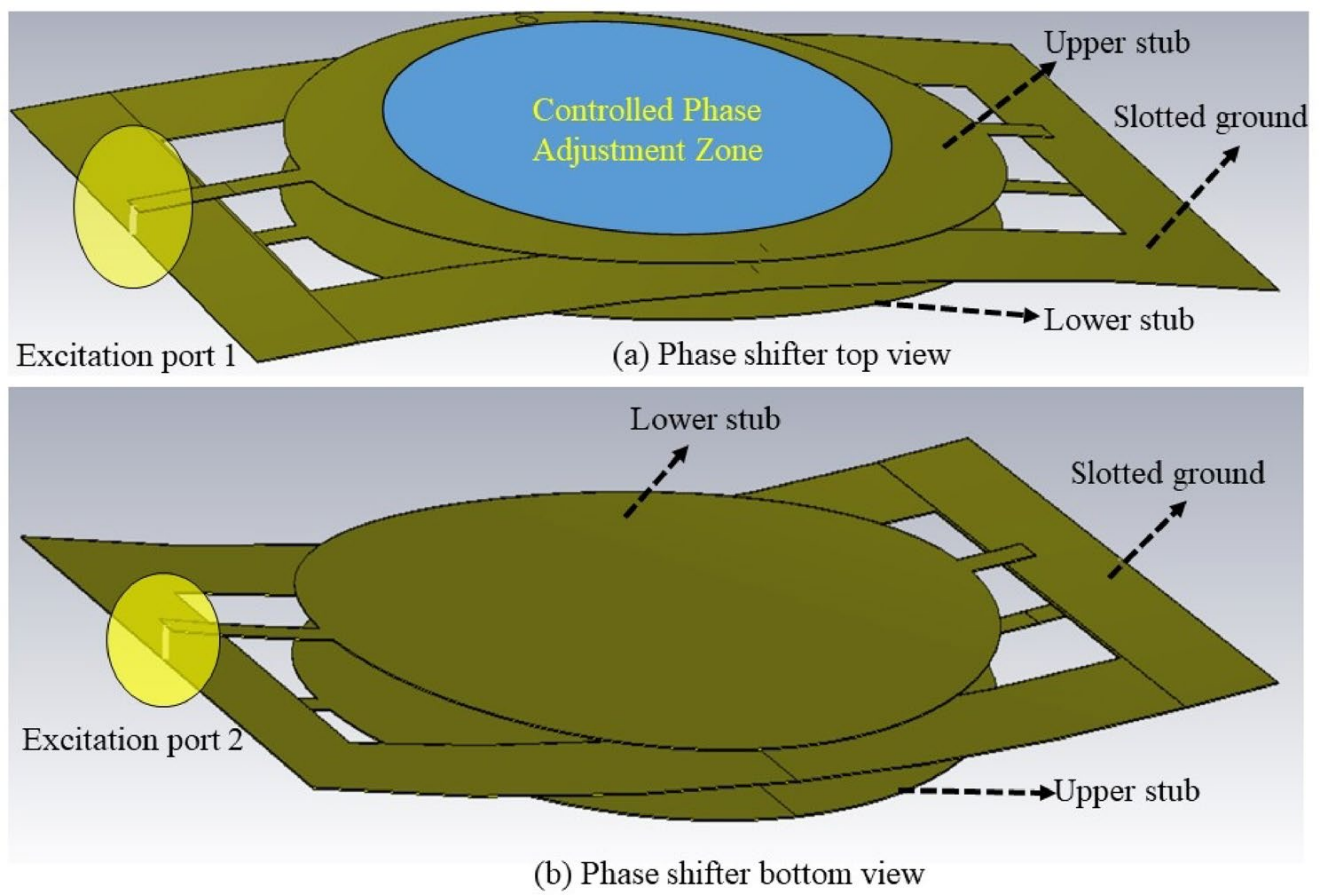
EM Full wave simulation model of the conformal µMP-AC phase shifter bent on cylindrical surface with radius 100 mm. (**a**) top view with controlled phase adjustment zone and excitation port 1 (**b**) bottom view with excitation port 2.

The conformal implementation of the µMP-AC phase shifter preserves the same geometric layout as the planar case while being bent onto the cylindrical surface (*R* = 100 mm). The corresponding top and bottom views of the conformal µMP-AC phase shifter are illustrated in Fig. 9, highlighting the structural conformity and mechanical feasibility of the proposed design on curved surfaces.

**Fig. 10:**
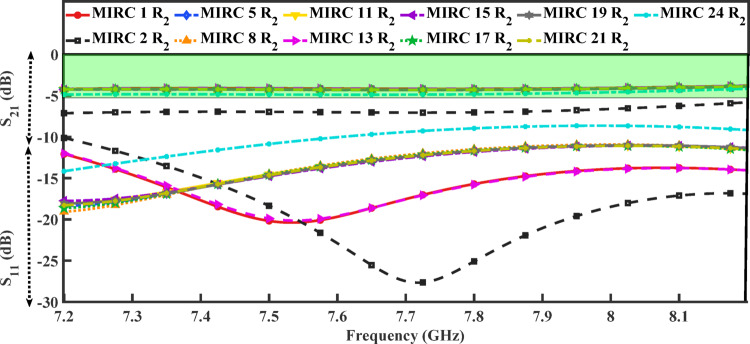
Conformal µMP-AC phase shifter reflection $\:\left|{S}_{11}\right|$ and transmission $\:\left|{S}_{21}\right|$ coefficients in dB for sequential activation of MIRCs (1–24) in reference array at $\:{\mathrm{R}}_{2}=6\:\mathrm{m}\mathrm{m}$.

Like the planar configuration, the MIRCs are sequentially activated using a single small magnet placed beneath the lower substrate RT 5880. For all MIRC activations at R₂, the EM full wave simulated reflection coefficients satisfies $\:{\left|S\right|}_{11}\le\:-10\:\mathrm{d}\mathrm{B}$ across the entire operating frequency band 7.2–8.2 GHz, while the insertion loss remains better than ($\:\left|{S}_{21}\right|$ $\:<-5\:\mathrm{d}\mathrm{B})$ as shown in Fig. 10.

**Fig. 11:**
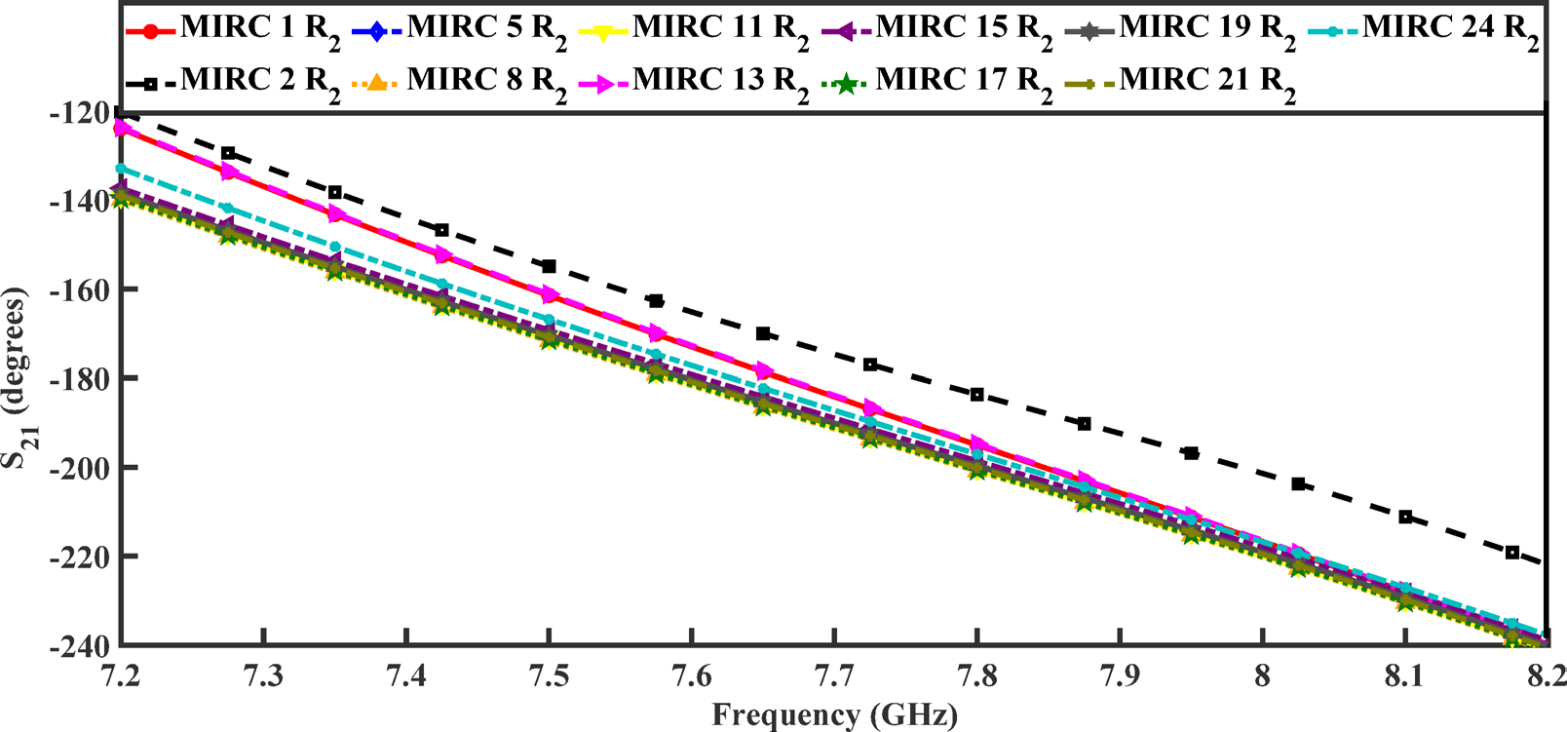
Conformal µMP-AC transmission phase response $\:{S}_{21}{\left(\right)}^{^\circ\:}$, for sequential activation of MIRCs (1–24) in the reference array at radius at $\:{\mathrm{R}}_{2}=6\:\mathrm{m}\mathrm{m}$.

**Case 1: MIRCs activation at R**_**2**_: The phase behavior of the conformal µMP-AC phase shifter is first explored for MIRC activations at radius R₂. In addition to maintaining good impedance matching and moderate insertion loss transmission, the corresponding phase responses demonstrate linear behavior (shown in Fig. 11) across the full frequency range of 7.2–8.2 GHz, indicating stable and broadband phase control under conformal conditions.

Importantly, the phase linearity observed in the planar configuration is preserved when the phase shifter is bent onto a cylindrical surface with a radius of 100 mm. This confirms that the proposed µMP-AC phase shifter operates effectively on both planar and conformal geometries without degradation in phase linearity or bandwidth performance using a single magnet.

**Case 2: MIRC Activation at R**_**2**_: The conformal µMP-AC phase shifter is next investigated for MIRC activations at the larger radius R₃ = 9 mm on a cylindrical surface with a radius of 100 mm. Phase tuning is achieved by sequentially activating individual MIRCs using a single tiny magnet placed beneath the lower Rogers RT5880 substrate.

**Fig. 12:**
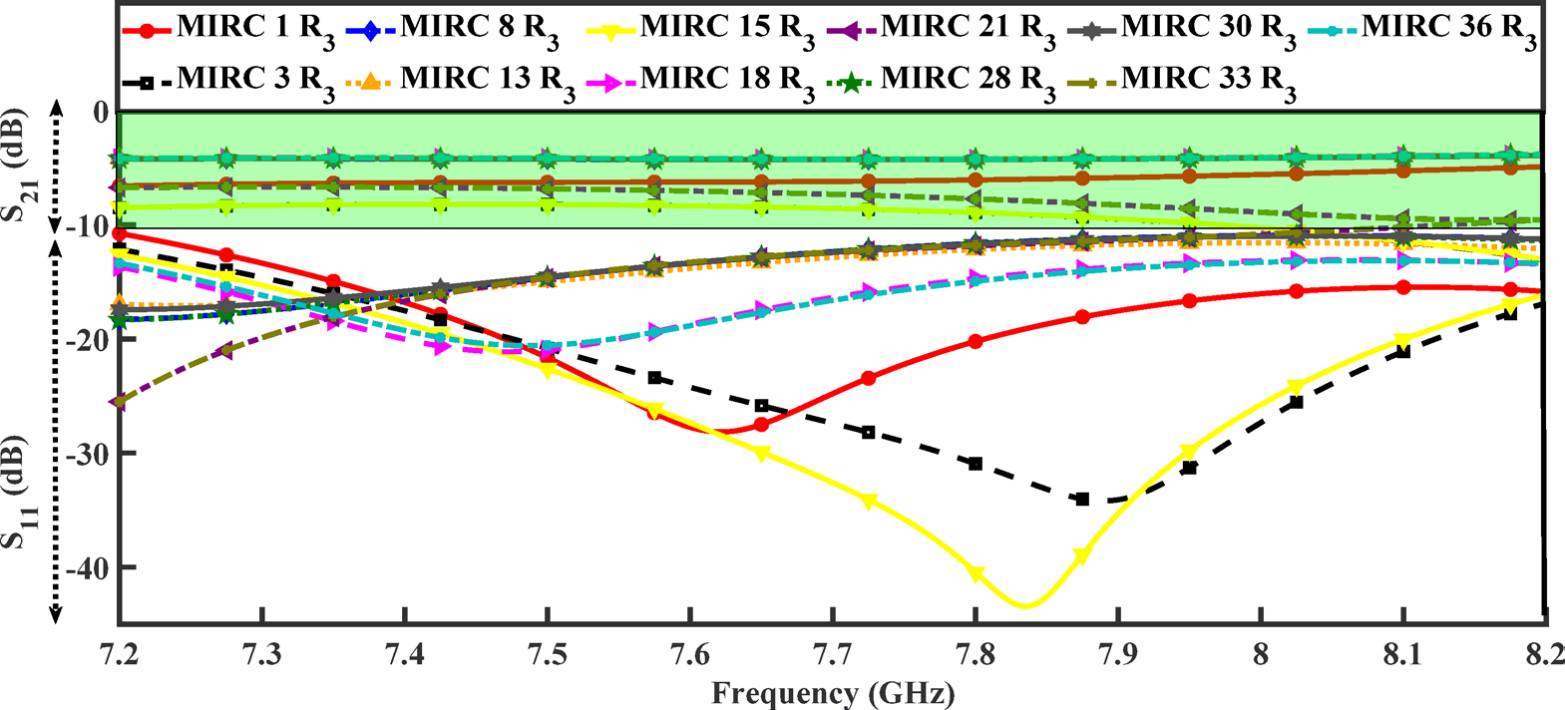
Conformal µMP-AC phase shifter reflection $\:\left|{S}_{11}\right|$ and transmission $\:\left|{S}_{21}\right|$ coefficients in dB for sequential activation of MIRCs (1–36) in reference array at $\:{\mathrm{R}}_{3}=9\:\mathrm{m}\mathrm{m}$.

**Fig. 13:**
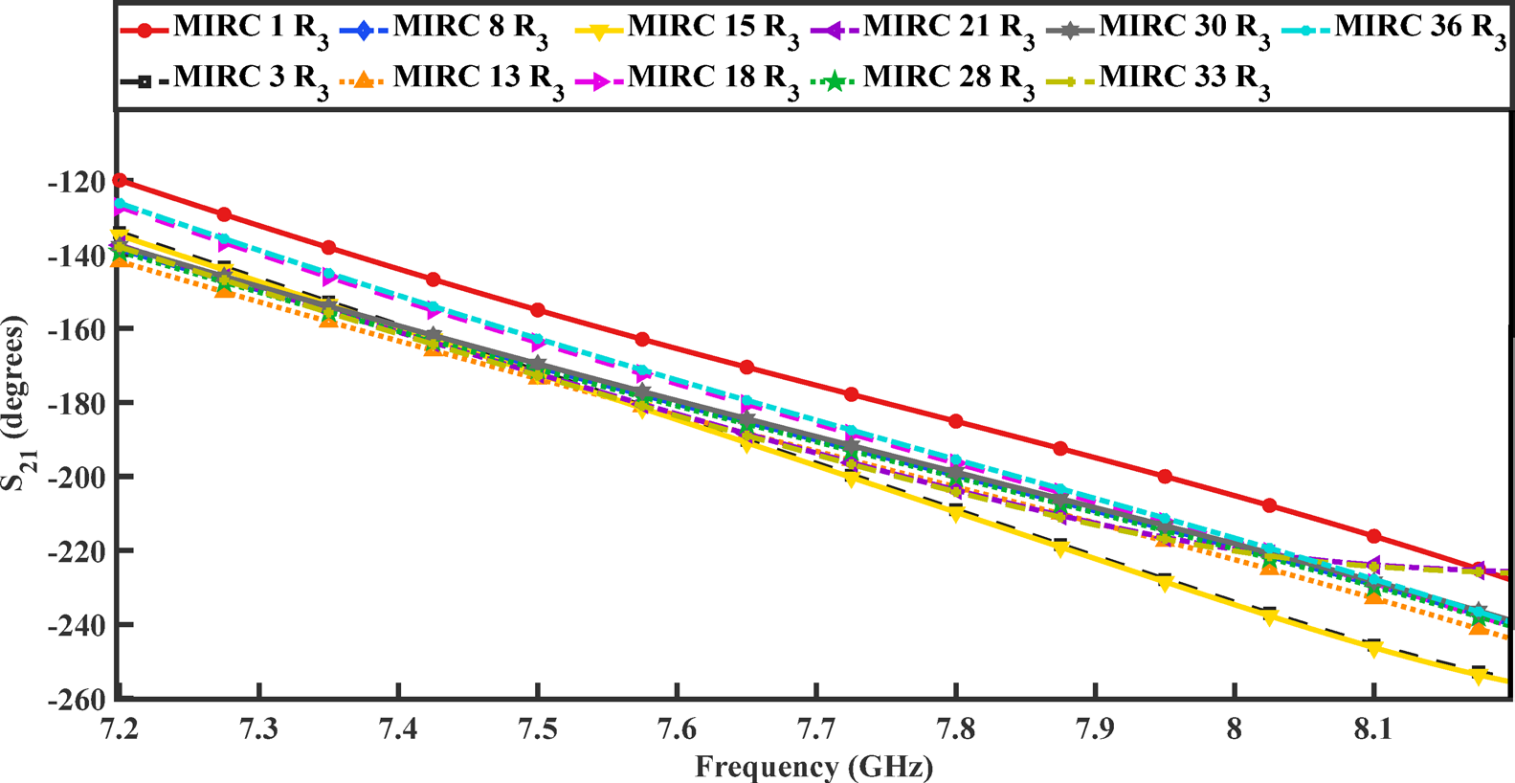
Conformal µMP-AC transmission phase response $\:{S}_{21}{\left(\right)}^{^\circ\:}$, for sequential activation of MIRCs (1–36) in the reference array at radius at $\:{\mathrm{R}}_{3}=9\:\mathrm{m}\mathrm{m}$.

As shown in Fig. 12, the structure maintains good impedance matching over the entire 7.2–8.2 GHz band, with $\:{\left|S\right|}_{11}\le\:-10\:\mathrm{d}\mathrm{B}$ for all MIRC activations, while the insertion loss remains better than ($\:\left|{S}_{21}\right|$ $\:<-5\:\mathrm{d}\mathrm{B})$. The corresponding transmission phase responses ∠S21 (Fig. 13) shows a nearly linear frequency dependence across the operating band. At the center frequency of 7.7 GHz, the phase varies approximately from − 200° to 160°, yielding a maximum relative phase shift. These results confirm that activating MIRCs at R₃ provides a larger phase shift compared to R₂ while preserving broadband operation and phase linearity under conformal bending (*R* = 100 mm). This demonstrates that the proposed µMP-AC phase shifter is well suited for compact conformal radar applications, enabling the generation of different linear phase states using a single movable magnet.

To further validate the conformal performance, a quantitative comparison between planar and cylindrical configurations (*R* = 100 mm) is presented in Table 1. The comparison includes key performance metrics such as insertion loss, return loss, phase range, phase error, and bandwidth. The results indicate that the proposed µMP-AC phase shifter maintains consistent phase linearity, impedance matching, and moderate insertion loss under bending conditions. It can be observed that all major performance parameters remain largely unchanged when transitioning from planar to conformal geometry. This demonstrates that conformal implementation does not significantly degrade electromagnetic performance, confirming its suitability for conformal phased array and radar applications.

**Table 1:** Comparison between planar and conformal µMP-AC phase shifter.

Parameter	Planar configuration	Conformal configuration (*R* = 100 mm)
Insertion Loss (dB)	< 5 dB	< 5 dB
Return Loss (dB)	~ 37.24 dB	Comparable (< -30 dB)
Phase Range (°)	0° – 40°	0° – 40°
Phase Error (°)	~ 1.2°	~ 1–2°
Bandwidth (GHz)	7.2–8.2 GHz	7.2–8.2 GHz

In the next section, the proposed conformal µMP-AC phase shifter is validated through circuit-level simulations using Advanced Design System (ADS) software.

## Conformal µMP-AC phase shifter circuit-level modeling and validation

**Fig. 14:**
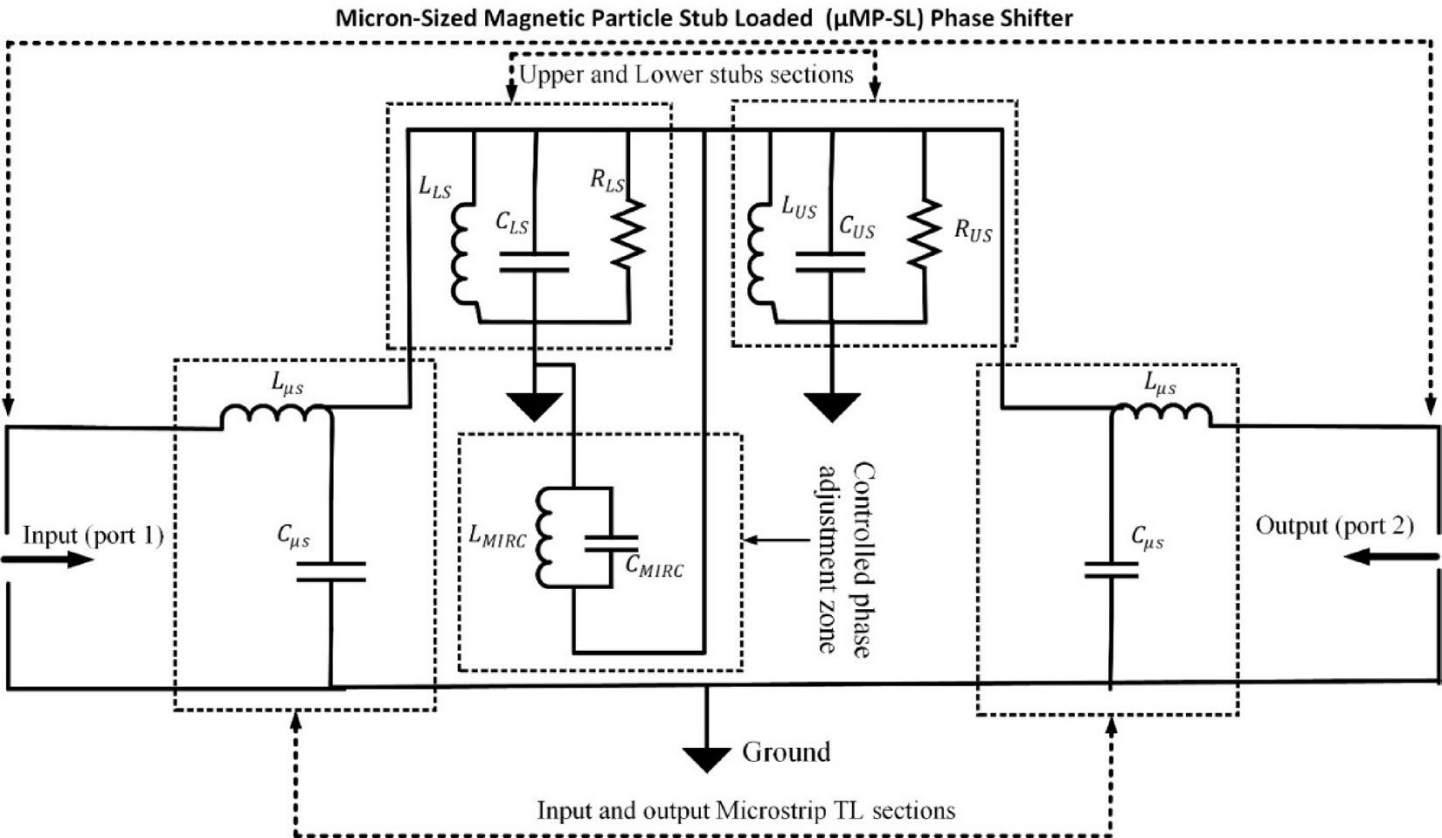
Conformal µMP-AC phase shifter equivalent circuit using advanced design.

In this section, the proposed conformal µMP-AC phase shifter full-wave model, presented in the previous section, is validated using an equivalent circuit-level simulation model in Advanced Design System (ADS) software. The equivalent circuit is adapted from^40^, with modifications to include the small inductance introduced by magnetic particles in the cavities, represented as $\:{\mathrm{L}}_{\mathrm{M}\mathrm{I}\mathrm{R}\mathrm{C}}$ as shown in Fig. 1. In the equivalent circuit, shown in Fig. 14, the microstrip transmission line sections consist of series inductance $\:{\mathrm{L}}_{{\upmu\:}\mathrm{s}}$ and parallel capacitance $\:{\mathrm{C}}_{{\upmu\:}\mathrm{s}}$are placed at input and output. The magnetically controlled Phase Adjustment Zone (CPAZ) is subsequently introduced, comprising two resonating circular stubs (upper and lower) together with the MIRC inductance $\:{\mathrm{L}}_{\mathrm{M}\mathrm{I}\mathrm{R}\mathrm{C}}$ and capacitance $\:{\mathrm{C}}_{\mathrm{M}\mathrm{I}\mathrm{R}\mathrm{C}}$. The lower stub circuit elements are represented by $\:{\mathrm{L}}_{\mathrm{L}\mathrm{S}}$,$\:\:{\mathrm{R}}_{\mathrm{L}\mathrm{S}}$ and $\:{\mathrm{C}}_{\mathrm{L}\mathrm{S}}$ while the upper stubs are represented by $\:{\mathrm{L}}_{\mathrm{U}\mathrm{S}}$,$\:\:{\mathrm{R}}_{\mathrm{U}\mathrm{S}}$ and $\:{\mathrm{C}}_{\mathrm{U}\mathrm{S}}$. The 50 Ω termination points relate to series inductance $\:{\mathrm{L}}_{{\upmu\:}\mathrm{s}}$on both sides.

System (ADS) Software.

Further, the magnetic particle–loaded region called CPAZ is modeled as a lumped inductive–capacitive network whose effective parameters ($\:{\mathrm{L}}_{\mathrm{M}\mathrm{I}\mathrm{R}\mathrm{C}},{\mathrm{C}}_{\mathrm{M}\mathrm{I}\mathrm{R}\mathrm{C}})$ vary with magnet position, capturing the magnetically induced phase shift.

To further strengthen the theoretical foundation, the particle-loaded CPAZ region is modeled using an effective medium approach, where the micron-sized magnetic particles embedded in the cavities are represented by equivalent lumped parameters. The alignment of particles under an external magnetic field alters the effective permittivity of the cavity region, which is captured in the circuit model through variations in the equivalent capacitance and inductance values. This approach establishes a direct link between the physical particle behavior and the circuit-level representation, enabling accurate prediction of the phase response. The variation of these parameters with magnet position reflects the tunable nature of the proposed phase shifter.

Furthermore, the circuit level modeling of each section (Fig. 14) of the proposed µMP-AC phase shifter is carried out using cascaded ABCD parameters, following the standard network analysis approach described in^38^. Each subsection of the phase shifter, including the microstrip transmission lines, resonating stubs, and the magnetically controlled Phase Adjustment Zone (CPAZ), is represented by its corresponding ABCD parameters in matrix form. These all-individual matrices are then cascaded to form the whole ABCD matrix of the proposed phase shifter. The resulting composite ABCD matrix is subsequently converted into S-parameters to extract the key performance metrics, like reflection coefficient (dB), insertion loss (dB), and transmission phase (degrees) response. The lumped-element values in the circuit model are iteratively optimized to achieve close agreement with the full-wave electromagnetic simulation results across the targeted operating frequency band of 7.2–8.2 GHz, thereby ensuring accurate circuit-level representation of the proposed structure.

***Case-1: microstrip transmission line***:

The input and output (Fig. 14) represent a Microstrip Transmission Line ($\:\mathrm{M}\mathrm{T}\mathrm{L})$ consists of $\:{L}_{\mu\:s}$ and $\:{C}_{\mu\:s}$,representing the upper and lower transmission lines. Using the cascaded approach of series impedance $\:{\mathrm{Z}}_{\mu\:s}$ and parallel admittance$\:\:{\mathrm{Y}}_{\mu\:s}$, the ABCD parameters of microstrip transmission line in terms of $\:{\mathrm{Z}}_{\mu\:s}$ and $\:{\mathrm{Y}}_{\mu\:s}$ are computed as^29^:

1 $$\:{\left(\begin{array}{cc}\mathrm{A}&\:\mathrm{B}\\\:\mathrm{C}&\:\mathrm{D}\end{array}\right)}_{\mathrm{M}\mathrm{T}\mathrm{L}}={\left\{\left(\begin{array}{cc}1&\:{\mathrm{Z}}_{{\upmu\:}\mathrm{s}}\\\:0&\:1\end{array}\right)\left(\begin{array}{cc}1&\:0\\\:{\mathrm{Y}}_{{\upmu\:}\mathrm{s}}&\:1\end{array}\right)\right\}}^{MTL\:lower}{\left\{\left(\begin{array}{cc}1&\:{\mathrm{Z}}_{{\upmu\:}\mathrm{s}}\\\:0&\:1\end{array}\right)\left(\begin{array}{cc}1&\:0\\\:{\mathrm{Y}}_{{\upmu\:}\mathrm{s}}&\:1\end{array}\right)\right\}}^{MTL\:upper}$$

Similarly, the ABCD parameters for CPAZ circular stubs (CPAZ-CS) are defined by:

2 $$\:{\left(\begin{array}{cc}\mathrm{A}&\:\mathrm{B}\\\:\mathrm{C}&\:\mathrm{D}\end{array}\right)}_{\mathrm{C}\mathrm{P}\mathrm{A}\mathrm{Z}-\mathrm{C}\mathrm{S}}={\left\{\left(\begin{array}{cc}1&\:0\\\:\left(\frac{1}{{R}_{S}}\right)+j\omega\:{C}_{S}-\frac{j}{j\omega\:{L}_{S}}&\:1\end{array}\right)\right\}}^{LS}{\left\{\left(\begin{array}{cc}1&\:0\\\:\left(\frac{1}{{R}_{S}}\right)+j\omega\:{C}_{S}-\frac{j}{j\omega\:{L}_{S}}&\:1\end{array}\right)\right\}}^{US}$$

The LS stands for Lower Stub while the US for Upper Stub in Eq. (2). Further the MIRC section ABCD parameters are defined by:

3 $$\:{\left(\begin{array}{cc}\mathrm{A}&\:\mathrm{B}\\\:\mathrm{C}&\:\mathrm{D}\end{array}\right)}_{\mathrm{M}\mathrm{I}\mathrm{R}\mathrm{C}}=\left(\begin{array}{cc}1&\:0\\\:j\omega\:{C}_{d}-\frac{j}{j\omega\:{L}_{\mathrm{M}\mathrm{I}\mathrm{R}\mathrm{C}}}&\:1\end{array}\right)$$

In Eq. (3), the $\:{\mathrm{L}}_{\mathrm{M}\mathrm{I}\mathrm{R}\mathrm{C}}$ is defined by^37^.

4 $$\:{L}_{MIRC}=\frac{{\mu\:}_{0}}{2\pi\:}\left[h.\mathrm{ln}\left(\frac{h+\sqrt{{r}^{2}+{h}^{2}}}{r}\right)+\frac{3}{2}\left(r-\sqrt{{r}^{2}+{h}^{2}}\right)\right]+\frac{{Z}_{stub}\sqrt{{\epsilon\:}_{re}}}{{c}_{0}}{L}_{{s}^{{\prime\:}}}+\:\:\:\:\:\:\:\:\:\:\:\:\:\:\:\:\:\:\:\:\:\:\:\:\:\:\:\:\:\:\:\:\:\:\:\:\:\:\frac{{Z}_{3}\sqrt{{\epsilon\:}_{re}}}{{c}_{0}}{W}_{{s}^{{\prime\:}}}$$

The final ABCD matrix of the conformal µMP-AC phase shifter is computed by cascading the ABCD matrices of the individual components:

5 $$\:{\left(\begin{array}{cc}\mathrm{A}&\:\mathrm{B}\\\:\mathrm{C}&\:\mathrm{D}\end{array}\right)}_{{\upmu\:}\mathrm{M}\mathrm{P}-\mathrm{A}\mathrm{C}}={\left(\begin{array}{cc}\mathrm{A}&\:\mathrm{B}\\\:\mathrm{C}&\:\mathrm{D}\end{array}\right)}_{\mathrm{M}\mathrm{T}\mathrm{L}}{\left(\begin{array}{cc}\mathrm{A}&\:\mathrm{B}\\\:\mathrm{C}&\:\mathrm{D}\end{array}\right)}_{\mathrm{C}\mathrm{P}\mathrm{A}\mathrm{Z}-\mathrm{C}\mathrm{S}}{\left(\begin{array}{cc}\mathrm{A}&\:\mathrm{B}\\\:\mathrm{C}&\:\mathrm{D}\end{array}\right)}_{\mathrm{M}\mathrm{I}\mathrm{R}\mathrm{C}}{\left(\begin{array}{cc}\mathrm{A}&\:\mathrm{B}\\\:\mathrm{C}&\:\mathrm{D}\end{array}\right)}_{\mathrm{M}\mathrm{T}\mathrm{L}}$$

From the final ABCD matrix, the scattering parameters (S-parameters) can be calculated using^38^:

6 $$\:{\left[\mathrm{S}\right]}_{{\upmu\:}\mathrm{M}\mathrm{P}-\mathrm{A}\mathrm{C}}=\left(\begin{array}{cc}\frac{\mathrm{A}+\frac{\mathrm{B}}{{\mathrm{Z}}_{\mathrm{O}}}-\mathrm{C}{\mathrm{Z}}_{\mathrm{O}}-\mathrm{D}}{\mathrm{A}+\frac{\mathrm{B}}{{\mathrm{Z}}_{\mathrm{O}}}+\mathrm{C}{\mathrm{Z}}_{\mathrm{O}}+\mathrm{D}}&\:\frac{2(\mathrm{A}\mathrm{D}-\mathrm{B}\mathrm{C})}{\mathrm{A}+\frac{\mathrm{B}}{{\mathrm{Z}}_{\mathrm{O}}}+\mathrm{C}{\mathrm{Z}}_{\mathrm{O}}+\mathrm{D}}\\\:\frac{2}{\mathrm{A}+\frac{\mathrm{B}}{{\mathrm{Z}}_{\mathrm{O}}}+\mathrm{C}{\mathrm{Z}}_{\mathrm{O}}+\mathrm{D}}&\:\frac{-\mathrm{A}+\frac{\mathrm{B}}{{\mathrm{Z}}_{\mathrm{O}}}-\mathrm{C}\mathrm{Z}0+\mathrm{D}}{\mathrm{A}+\frac{\mathrm{B}}{{\mathrm{Z}}_{\mathrm{O}}}+\mathrm{C}{\mathrm{Z}}_{\mathrm{O}}+\mathrm{D}}\end{array}\right)$$

Here the $\:{Z}_{o}$ is the characteristics impedance of the phase shifter. The Eq. (6) can be simplified and denoted in standard S-matrix form suchas:

7 $$\:\:\:\left(\mathrm{S}\right)=\left(\begin{array}{cc}{\mathrm{S}}_{11}&\:{\mathrm{S}}_{12}\\\:{\mathrm{S}}_{21}&\:{\mathrm{S}}_{22}\end{array}\right)\:\:$$

The phase shift through the $\:{\upmu\:}\mathrm{M}\mathrm{P}-\mathrm{A}$L phase shifter is obtained from:$\:\left(7\right)$

8 $$\:{{\upphi\:}}_{{\mathrm{S}}_{21}\:}\left(\mathrm{d}\mathrm{e}\mathrm{g}\right)=\mathrm{p}\mathrm{h}\mathrm{a}\mathrm{s}\mathrm{e}\:\mathrm{o}\mathrm{f}\:{\mathrm{S}}_{21}$$

9 $$\:\mathrm{I}\mathrm{n}\mathrm{s}\mathrm{e}\mathrm{e}\mathrm{t}\mathrm{i}\mathrm{o}\mathrm{n}\:\mathrm{L}\mathrm{o}\mathrm{s}\mathrm{s}\:\left(\mathrm{d}\mathrm{B}\right)=20{\mathrm{log}}_{10}\left|{\mathrm{S}}_{21}\right|\:$$

10 $$\:{\Gamma\:}\left(\mathrm{d}\mathrm{B}\right)=20{\mathrm{log}}_{10}\left|{\mathrm{S}}_{11}\right|$$

**Fig. 15:**
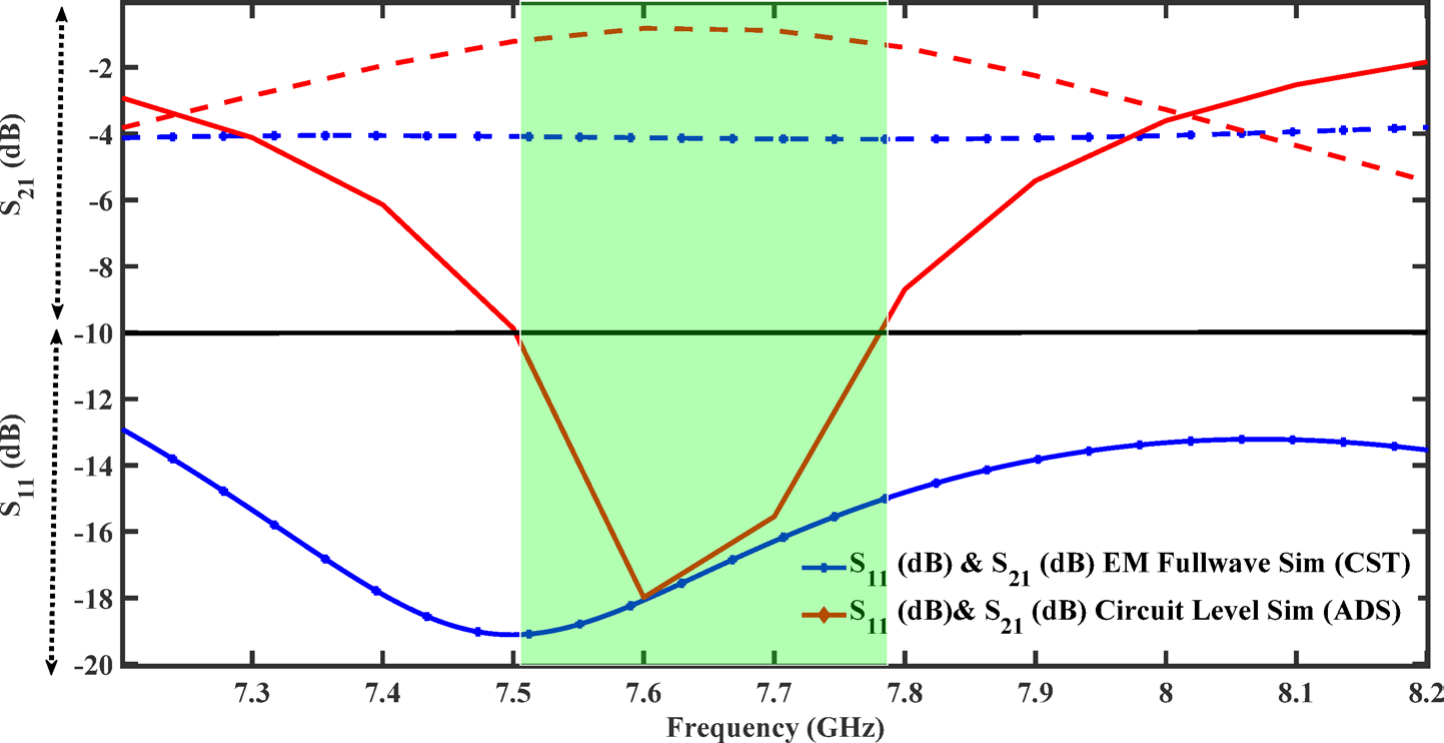
Conformal µMP-AC phase shifter reflection $\:\left|{S}_{11}\right|$ and transmission $\:\left|{S}_{21}\right|$ coefficients validation using CST and ADS software.

Expression (8) defines the transmission phase shift (deg) between input port 1 and output port 2 of the proposed conformal µMP-AC phase shifter, as illustrated in Figs. 9 and 14. This expression captures the phase evolution of the transmitted signal resulting from the combined effects of the microstrip transmission lines, aperture-coupled resonant stubs, and the magnetically controlled Phase Adjustment Zone (CPAZ). Equations (9) and (10) define the key amplitude performance metrics: the insertion loss and the reflection coefficient, both expressed in decibels (dB) (Fig. 15). Together, they provide a complete assessment of the phase shifter’s magnitude characteristics. Specifically, Eq. (9) quantifies the signal attenuation through the device, while Eq. (10) evaluates the impedance matching at the input port.

**Fig. 16:**
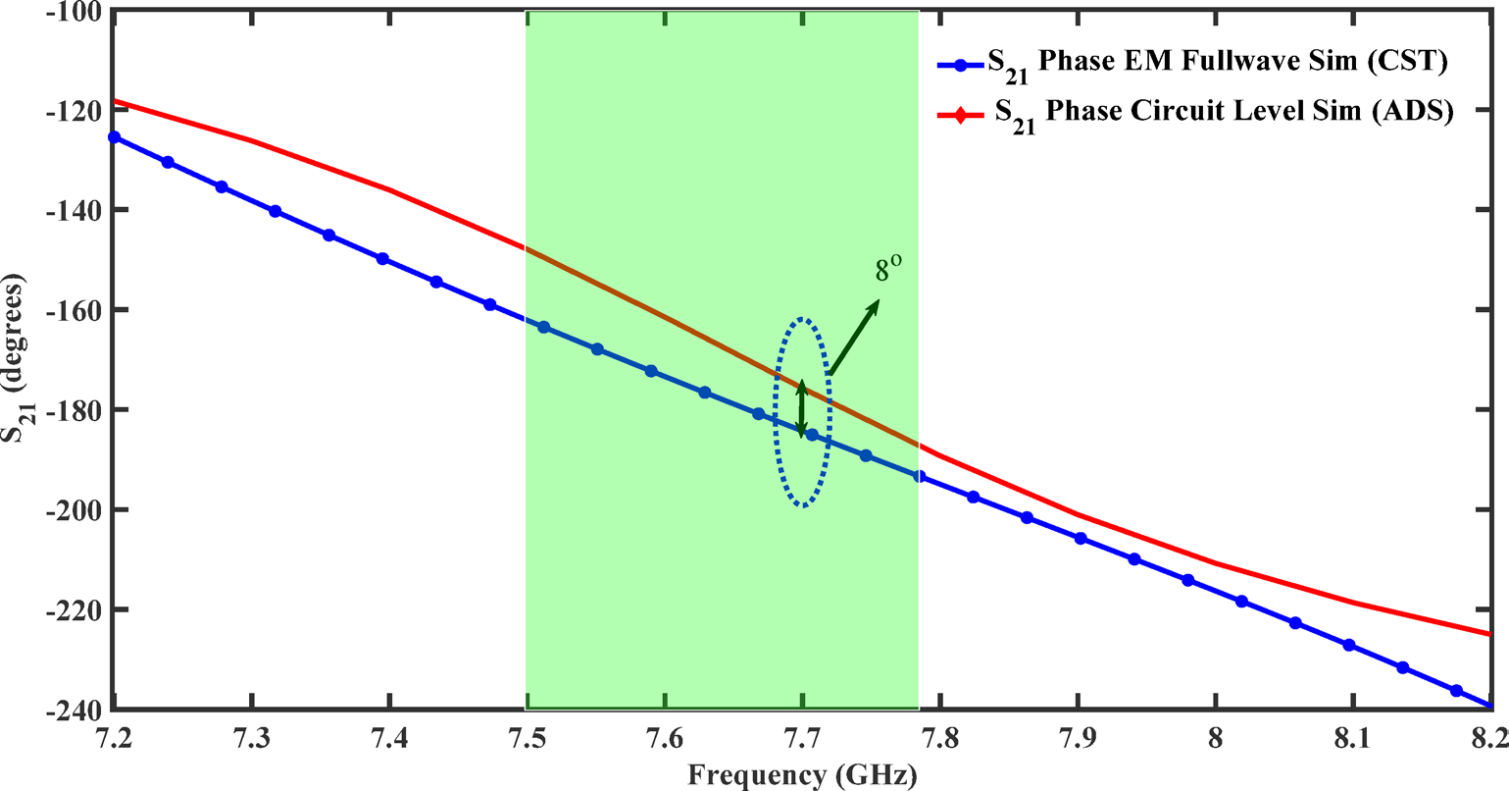
Conformal µMP-AC transmission phase response $\:{S}_{21}{\left(\right)}^{^\circ\:}$ validation using CST and ADS software.

For the target bandwidth of $\:{S}_{11}$ $\:=(7.2-8.2)\:\mathrm{G}\mathrm{H}\mathrm{z}$, with an insertion loss insertion loss $\:{S}_{21}=$ $\:-2\:\mathrm{t}\mathrm{o}-5\:\mathrm{d}\mathrm{B}$ and a linear phase response $\:\angle\:{S}_{21}=$ $\:{-240}^{^\circ\:}$ to $\:{-120}^{^\circ\:}$ (Fig. 16), the equivalent ABCD parameters of the µMP-AC phase shifter are determined using Eqs. (8) and (9). Based on these parameters, the required inductance and capacitance values for each section are calculated following the methodology in^29^. The resulting parameters values ($\:{\mathrm{L}}_{{\upmu\:}\mathrm{s}}$=0.98 nH, $\:{\mathrm{C}}_{{\upmu\:}\mathrm{s}}$=1.2 pF, $\:{\mathrm{L}}_{\mathrm{S}}=0.21\:\mathrm{n}\mathrm{H}$, $\:{\mathrm{C}}_{\mathrm{S}}=0.6\:\mathrm{p}\mathrm{F}$, $\:{\mathrm{L}}_{\mathrm{M}\mathrm{I}\mathrm{R}\mathrm{C}}=10\:\mathrm{n}\mathrm{H}$ and $\:{\mathrm{C}}_{\mathrm{M}\mathrm{I}\mathrm{R}\mathrm{C}}$ =1.05 pF) are computed using Eqs. (3–10) in the MATLAB environment and subsequently implemented in Advanced Design System (ADS) software using the configuration shown in Fig. 14. The simulation results, presented in Fig. 15 (reflection coefficients and insertion loss) demonstrate excellent agreement with the design specifications. Similarly, the achieved controlled phase response within the desired bandwidth (7.2-8.2) GHz shows excellent agreement with the ADS simulation results, with a minimal phase error. as illustrated in Fig. 16. These results (Figs. 15 and 16) validate the full wave EM model (CST) and circuit level (ADS) simulation of the proposed conformal µMP-AC phase shifter in 3D EM model.

The next section presents experimental validation of the proposed µMP-AC phase shifter using VNA measurements in an in-house full anechoic chamber.

## Experimental Validation of µMP-AC phase shifter

**Fig. 17:**
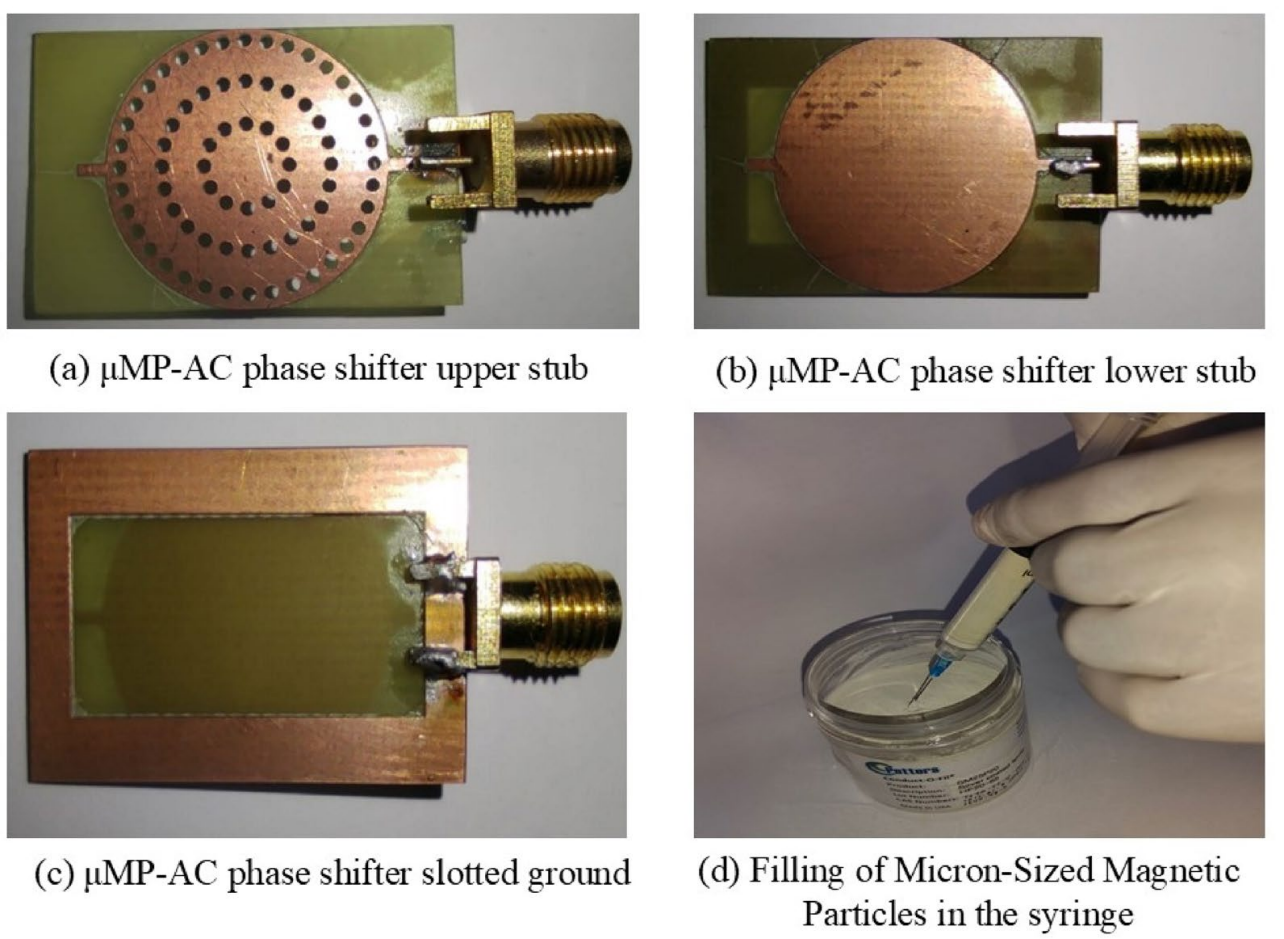
The µMP-AC Phase Shifter fabricated prototype and magnetic particles embedding.

**Fig. 18:**
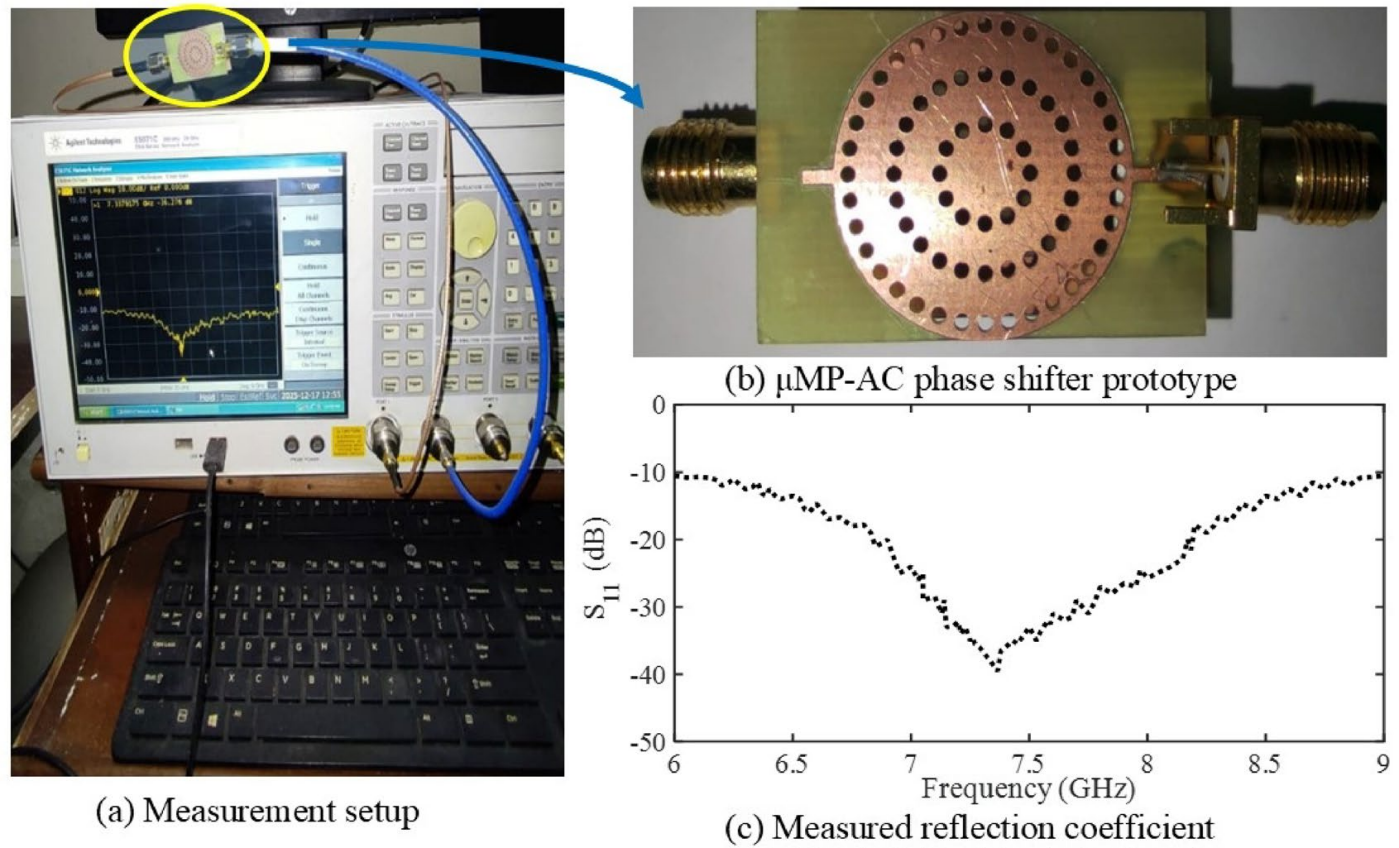
The measurement setup of the µMP-AC Phase Shifter fabricated prototype connected with VNA in chamber.

In this section, the proposed µMP-AC phase shifter is experimentally validated using measurements conducted in a fully calibrated in-house testing chamber. As a first step, the phase shifter is fabricated on a Rogers RT5880 substrate, as shown in Fig. 17. The fabricated upper and lower circular stubs are illustrated in Fig. 17a)and b, respectively. The slotted ground plane is shown in Fig. 17c. As a 2nd step, the magnetic particles^31^ required for phase shifting were loaded into a syringe, as illustrated in Fig. 17d. These particles were then selectively injected into the designated circular array cavities shown in Fig. 1 within the µMP-AC phase shifter. The filling process was carefully controlled to ensure uniform distribution and to enable the desired level of phase control. This selective cavity filling (at radial distance R_1_, R_2_, R_3_) is a key design feature that allows the linear phase shift response based on the presence and configuration of the magnetic material. In the last step 3, the fully fabricated prototype shown in Fig. 17, with partially filled magnetic cavities in circular arrays was connected to a Vector Network Analyzer (VNA) to perform reflection coefficients and phase measurements. Port 1 was used to measure the reflection coefficient $\:{|S}_{11}|$ (dB), while Port 2 was utilized to analyze the output signal for its phase shift characteristics in degrees. At an operating frequency of 7.3 GHz, the measured reflection coefficient $\:{|S}_{11}|$ was − 37.24 dB (Fig. 18c), indicating excellent impedance matching and minimum signal reflection, which closely matched the electromagnetic (EM) simulation results using CST Microwave Studio Suite 2025.

**Fig. 19:**
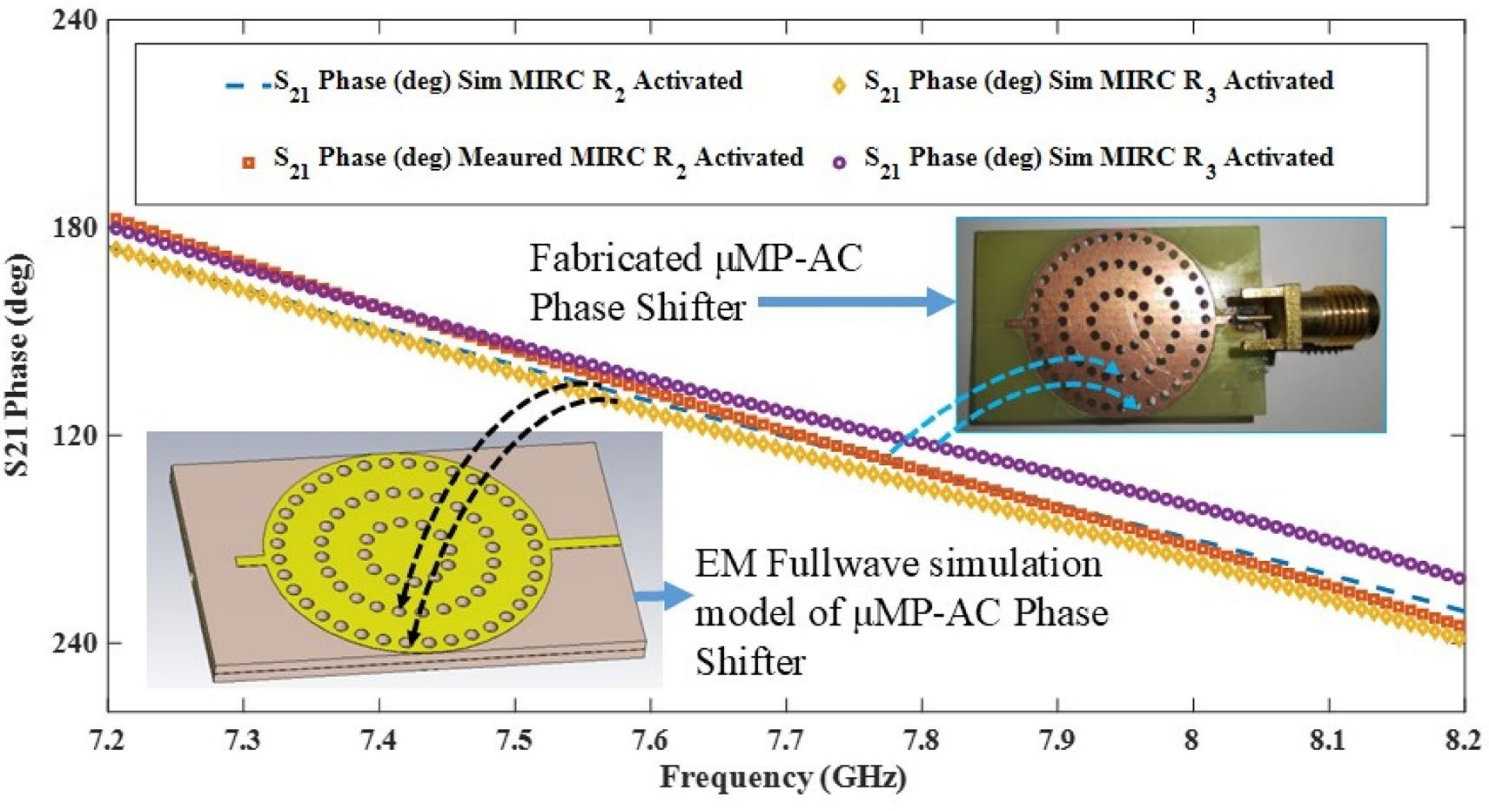
The measured and EM transmission phase response $\:{S}_{21}{\left(\right)}^{^\circ\:}$ of the µMP-AC phase shifter.

Further, the linear phase behavior is measured in the frequency range 7.2–8.2 GHz using VNA shown in Fig. 19. The electromagnetic (EM) simulated and measured phase response $\:{S}_{21}{\left(\right)}^{^\circ\:}$ shows excellent matching with minimum error of $\:{1.2}^{^\circ\:}$. Despite these small deviations in $\:{S}_{21}{\left(\right)}^{^\circ\:}$, the proposed µMP-AC phase shifter demonstrates linear phase control, which has been thoroughly validated across multiple simulators. The µMP-AC analytical modeling was performed using MATLAB environment, equivalent circuit-level simulations were carried out in ADS (Advanced Design System), full-wave EM simulations were executed using CST Microwave Studio Suite 2025, and final validation was performed through experimental measurements in a fully calibrated chamber using a Vector Network Analyzer (VNA). These excellent performance characteristics of the proposed µMP-AC phase shifter demonstrate its suitability for radar system applications on both planar and conformal surfaces. The ability to maintain moderate insertion loss, good impedance matching, and linear broadband phase response under surface curvature confirms the robustness of the proposed magnetically controlled phase-shifting mechanism. Furthermore, the use of a single movable tiny magnet enables a compact and DC-bias-free implementation, making the µMP-AC phase shifter a practical and efficient solution for conformal radar front-end architectures.

To further study the experimental reliability of the proposed µMP-AC phase shifter, measurement accuracy, repeatability, and loss mechanisms are presented. The measurements were conducted using a calibrated Vector Network Analyzer (VNA) in the In-house chamber to ensure high accuracy. The repeatability of the results (reflection coefficients, insertion loss and phase shifts) was verified by repositioning the external magnet multiple times at the same locations, yielding consistent phase responses with only minor observable variations. The observed insertion loss (dB) is attributed to a combination of dielectric loss from the Rogers RT5880 substrate, conductor loss in the metallic layers, and additional scattering and absorption losses introduced by the particle-loaded cavities.

Minor discrepancies between simulated and measured results are mainly due to fabrication tolerances and non-uniform particle distribution within the cavities. The measurement results confirm stable and reliable performance of the proposed phase shifter.

It should be noted that a full statistical repeatability analysis was not performed; however, repeated manual repositioning of the magnet produced consistent phase responses at the center frequency, confirming the stability of the proposed tuning mechanism.

## Conclusion

In this work, a conformal Micron-Sized Magnetic Particle Aperture-Coupled (µMP-AC) phase shifter is investigated, offering a passive, mechanically tunable solution with moderate insertion loss for wideband (7.2–8.2) GHz phase control, particularly for radar systems. The proposed design incorporates a circular Controlled Phase Adjustment Zone (CPAZ) in which Magnetic Induction–Responsive Cavities (MIRCs) are embedded in circular configurations at different radii of 3, 6, and 9 mm to achieve controlled phase response. The circular configuration at a radius of 3 mm consists of 12 MIRCs, while the configurations at 6 mm and 9 mm radii consist of 24 and 36 MIRCs, respectively. By magnetically activating these MIRCs using a single tiny magnet, linear and broadband phase responses of approximately $\:{0}^{^\circ\:}$,$\:\:{20}^{^\circ\:}$ and $\:{40}^{^\circ\:}$ are achieved on both planar and conformal cylindrical surfaces. The achievable phase range $\:{40}^{^\circ\:}$can be further extended either by integrating MIRCs at larger radii or by cascading multiple µMP-AC unit cells. This approach eliminates the need for external DC biasing circuits, thereby significantly simplifying the radar system architecture. The µMP-AC phase shifter design was thoroughly validated using full-wave 3D simulations in CST Microwave Studio 2025, proceeded to circuit-level analysis in Advanced Design System (ADS) and mathematical modeling in MATLAB, and concluded with experimental validation using a Vector Network Analyzer (VNA) in an in-house fully calibrated anechoic chamber. The excellent agreement observed among the full wave simulated, modeled, and measured results provides strong and comprehensive validation of the proposed phase shifter. The µMP-AC phase shifter is well suited for efficient, low-complexity beam-steering and conformal radar front-end systems due to its compact size, DC-bias-free operation, conformal capability, and single-magnet control.

## Data Availability

Data will be made available on reasonable request by the first author.
